# Growth Performance and Antioxidant Responses to Dietary Flavonoids in Swine: A Systematic Review and Meta-Analysis with Exploratory Dose–Time Relationships

**DOI:** 10.3390/antiox15080980

**Published:** 2026-08-07

**Authors:** Yi Xiao, Qiannan Han, Zhenglin Dong, Xizi Yang, Zhihao Xu, Kaiyu Hu, Zhihao Xuan, Guiping Liang, Weimin Jiang, Yinglin Peng, Yingying Liu

**Affiliations:** 1 Yuelushan Laboratory, Changsha 410128, China; xiaoyi@hunau.edu.cn (Y.X.); hanqiannan@stu.hunau.edu.cn (Q.H.); dongzhl@isa.ac.cn (Z.D.); yangxz12@hunaas.cn (X.Y.); guapideyouxiang@stu.hunau.edu.cn (Z.X.); 18773126229@stu.hunau.edu.cn (K.H.); xuanzhihao@stu.hunau.edu.cn (Z.X.); guipingliang@stu.hunau.edu.cn (G.L.); jwm111@hunaas.cn (W.J.); 2 College of Information and Intelligent Science and Technology, Hunan Agricultural University, Changsha 410128, China; 3 Hunan Institute of Animal and Veterinary Science, Changsha 410131, China

**Keywords:** flavonoids, pigs, growth performance, antioxidant capacity, meta-analysis

## Abstract

Dietary flavonoids are increasingly investigated in swine nutrition, but how supplementation level, intervention duration, and growth stage are associated with growth and antioxidant responses remains unclear. This PRISMA 2020 systematic review and meta-analysis evaluated dietary flavonoid supplementation in pigs. Twenty-eight randomized controlled trials were synthesized using three-level random-effects models to account for dependent effect sizes. Flavonoid supplementation was associated with increased average daily gain (ADG; SMD = 0.719, 95% CI: 0.428 to 1.009; $I^{2}=51.85\%$) and a lower feed-to-gain ratio (F/G; SMD = −0.708, 95% CI: −0.929 to −0.487; $I^{2}=32.25\%$), whereas the association with average daily feed intake (ADFI) was not statistically significant (SMD = 0.217, 95% CI: −0.101 to 0.535; $I^{2}=59.30\%$). Supplementation was also associated with increased superoxide dismutase (SMD = 0.907) and glutathione peroxidase activities (SMD = 0.666) and reduced malondialdehyde concentrations (SMD = −0.989), although heterogeneity was substantial for these outcomes ($I^{2}=96.47$–97.83%). The certainty of evidence was assessed as low for ADG and moderate for ADFI, F/G, and antioxidant outcomes under the adapted GRADE framework. Exploratory stage-specific dose–time meta-regression identified potential relationships between supplementation intensity and ADG in weaned pigs (35 effect sizes from 14 studies) and finishing pigs (12 effect sizes from four studies). In finishing pigs, intervention duration was associated with growth responses. However, this subgroup contained few independent studies, and intervention duration covaried with age, physiological stage, flavonoid source, dose, health status, and basal diet. This association therefore does not establish an independent causal effect of intervention duration.

## 1. Introduction

In modern intensive swine production systems, weaning stress induced by dietary transition, environmental changes, and social restructuring is a major factor contributing to intestinal barrier dysfunction and growth retardation in piglets. During weaning, metabolic alterations can induce excessive accumulation of reactive oxygen species (ROS), thereby triggering oxidative stress and inflammatory cascades and ultimately impairing nutrient digestion and absorption efficiency [1,2]. With the progressive restriction and prohibition of antibiotic growth promoters in many countries worldwide, the development of safe, sustainable, and effective alternatives has become an important focus in animal nutrition research [3].

Flavonoids, including quercetin and soy isoflavones, are naturally occurring plant-derived polyphenolic compounds. Their phenolic hydroxyl groups confer antioxidant activity through electron donation, free-radical scavenging, and metal-ion chelation [4,5,6,7]. Evidence suggests that flavonoids may enhance endogenous antioxidant defenses by regulating enzymes such as superoxide dismutase (SOD) and glutathione peroxidase (GSH-Px). These potential activities may contribute to reduced oxidative damage, maintenance of intestinal barrier integrity, and improved growth performance [8]. However, the reported effects of flavonoid supplementation in pigs remain inconsistent. Some studies have reported significant improvements in growth, whereas others have found weak or non-significant responses [9,10]. This heterogeneity may reflect differences in flavonoid subclass, supplementation dose, physiological stage, intervention duration, and metabolic status [11]. Most previous studies have focused on short-term effects under stress conditions, and quantitative evidence regarding dose- and duration-related responses across the full growth cycle remains limited. Biological responses to dietary flavonoids may vary according to supplementation intensity and exposure duration. Although higher supplementation levels may increase flavonoid availability, the activation of endogenous antioxidant defenses, regulation of inflammatory responses, and adaptation of intestinal functions are dynamic processes that may require sufficient exposure time. Responses may therefore vary with both supplementation level and intervention duration. However, whether dose and duration jointly explain variation in growth performance across physiological stages remains unclear. Moreover, differences in antioxidant responses among flavonoid subclasses and between systemic circulation and local tissues, such as the intestine and liver, have not been fully clarified [12].

Meta-analysis integrates findings from independent studies, quantifies overall intervention effects, and identifies potential sources of heterogeneity [13]. Following the PRISMA guidelines [14], we therefore systematically identified and synthesized randomized controlled trials to evaluate the potential effects of dietary flavonoids on growth performance and antioxidant capacity in pigs. Beyond estimating pooled effects, subgroup analyses compared responses across flavonoid subclasses, physiological stages, and tissue types. In addition, exploratory dose–time meta-regression analyses investigated potential associations of supplementation intensity and intervention duration with growth responses at different physiological stages. The findings may provide additional insight into the potential effects of dietary flavonoids and inform the development of antioxidant-based nutritional strategies for swine production [15,16].

## 2. Materials and Methods

### 2.1. Protocol and Reporting Standard

**Figure 1:**
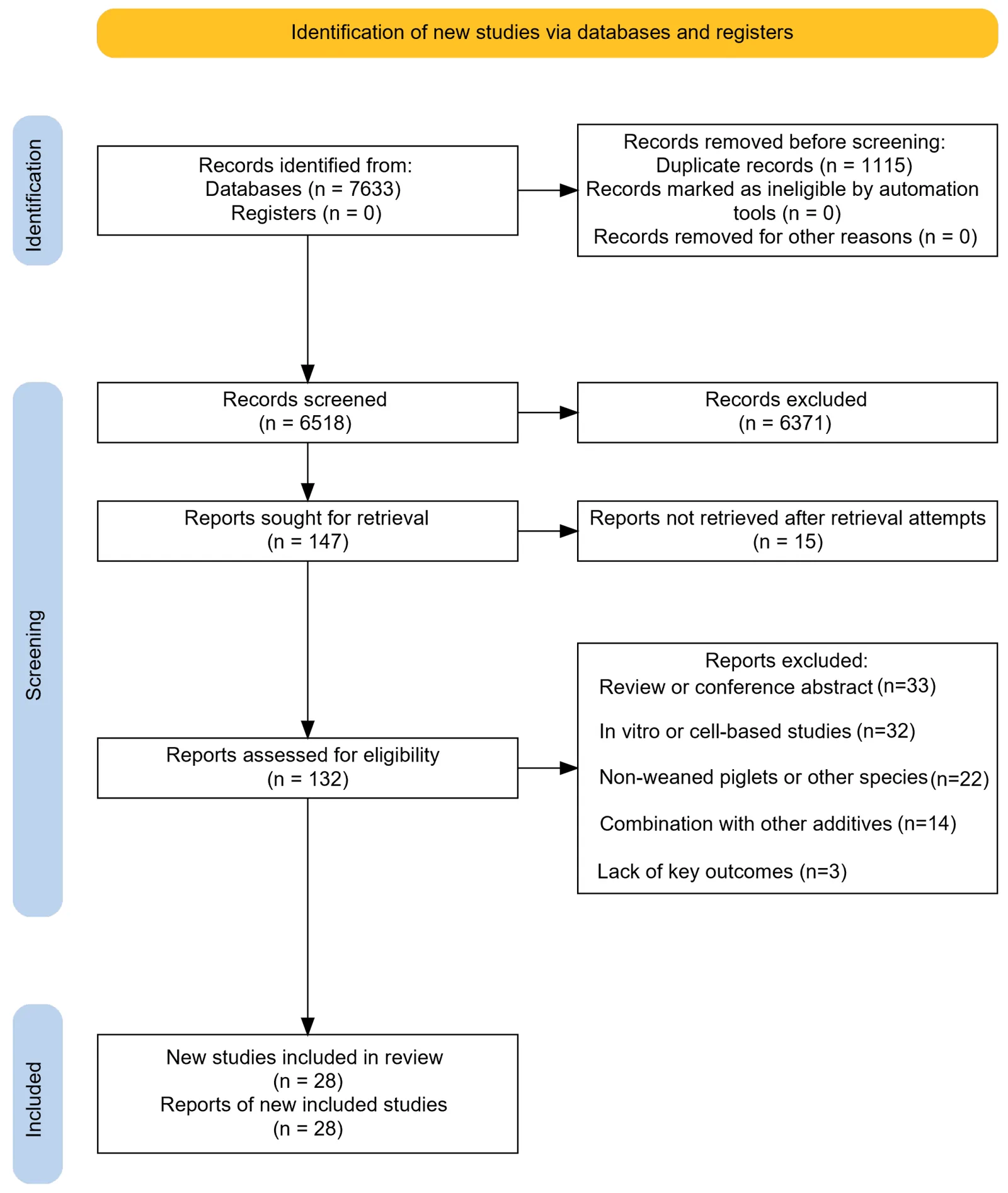
PRISMA flow diagram of the literature search and study selection process.

This systematic review and meta-analysis was conducted and reported in accordance with the Preferred Reporting Items for Systematic Reviews and Meta-Analyses (PRISMA) 2020 statement [17]. The study identification, screening, and inclusion process is illustrated in the PRISMA flow diagram (Figure 1). Procedures for literature screening, data extraction, and quality assessment were predefined before conducting the study to ensure methodological consistency.

### 2.2. Literature Search Strategy

To identify relevant studies comprehensively, two investigators independently searched PubMed, Web of Science, Scopus, and the China National Knowledge Infrastructure (CNKI). The final search was conducted on 31 January 2026. The search covered studies published from January 2001 to January 2026 and imposed no language restrictions. The strategy combined controlled-vocabulary and free-text terms in three domains: flavonoid compounds (e.g., flavonoids and quercetin), pigs (e.g., pigs, swine, and piglets), and outcomes (e.g., growth performance and antioxidant capacity). The reference lists of relevant reviews and included studies were also screened manually to identify eligible studies that the database searches might have missed. Gray literature, including conference abstracts, dissertations, and unpublished reports, was not searched. Additional attempts were made to obtain the full texts of potentially eligible reports that were not directly accessible. Reports that remained unavailable were recorded as not retrieved and were not assessed for eligibility.

### 2.3. Eligibility Criteria

The eligibility criteria were established according to the PICOS framework [18]:

- **Participants:** Pigs at various production stages. For subgroup analyses, the animals were classified into weaned piglets, growing pigs, or finishing pigs according to the original study descriptions.
- **Intervention:** Experimental groups received basal diets supplemented with flavonoid-containing preparations, including plant-derived extracts or purified flavonoid compounds. Studies were required to report clear supplementation information (e.g., mg/kg, g/t, %, mg/d, mL/t, or other identifiable dosing schemes). Studies involving explicit co-interventions with other functional additives (e.g., antibiotics, probiotics, or enzymes), in which the independent effects of flavonoid supplementation could not be determined, were excluded.
- **Comparison:** Control groups received basal diets without flavonoid supplementation.
- **Outcomes:** Studies were required to report at least one of the following outcomes: average daily gain (ADG), average daily feed intake (ADFI), feed-to-gain ratio (F/G), SOD, MDA, or GSH-Px.
- **Study Design:** Randomized controlled trials (RCTs).

For studies using plant-derived flavonoid extracts, the intervention was considered eligible when the extract was the primary dietary supplement investigated in the original study. However, because plant extracts may contain bioactive compounds other than flavonoids, the observed effects cannot be attributed exclusively to flavonoids. Potential contributions from non-flavonoid components were therefore considered when interpreting the findings. The exclusion criteria were as follows: (1) in vitro studies, non-porcine animal experiments, conference abstracts, or review articles; (2) studies with incomplete outcome data (e.g., missing standard deviation [SD] or standard error of the mean [SEM]) for which raw data could not be obtained from the corresponding authors; and (3) duplicate publications, for which only the most comprehensive dataset was retained.

### 2.4. Data Extraction

Two investigators independently screened the literature and extracted data using a prespecified standardized form. Disagreements were resolved through discussion or consultation with a third reviewer. When a study included multiple dose groups, multiple treatment groups sharing a common control group, or outcomes measured in different sample sources, each comparison was retained as a separate effect size to preserve the available information. The original control-group sample size was retained when calculating effect sizes rather than divided among comparisons. The following information was extracted: first author, publication year, pig growth stage, flavonoid subclass, supplementation dose or level, intervention duration, means and standard deviations (SDs) for each outcome (ADG, ADFI, F/G, SOD, MDA, and GSH-Px), and sample sources (e.g., serum, plasma, or tissue). The sample size (*N*) was defined as the number of statistical units (individual pigs or pens) reported in the original studies. The experimental unit was determined from the original study design. For pen-based experiments, the pen was treated as the experimental unit; when individual animals were the experimental units, the reported number of animals was retained. For studies reporting only the standard error of the mean (SEM), the SD was calculated as follows:

(1) $$\mathrm{SD}=\mathrm{SEM}\times \sqrt{N}$$

where *N* is the sample size of the corresponding treatment or control group.

### 2.5. Statistical Analysis

All statistical analyses were performed and all figures were generated using R software (version 4.4.3) with the metafor package [19]. The significance threshold was set at p<0.05.

#### 2.5.1. Effect Size Calculation and Model Selection

Because some studies contributed multiple supplementation levels or measurements from different sample sources, these effect sizes were not statistically independent. Therefore, three-level random-effects models were fitted using the rma.mv() function in the metafor package [20]. Effect sizes were nested within studies to account for within-study dependence and between-study heterogeneity. The model partitioned variance into sampling variance at the effect-size level (level 1), variation among multiple effect sizes within the same study (level 2), and between-study heterogeneity (level 3). The random-effects structure was specified as ^~^1 | Study_ID/Effect_ID.

Because correlations among effect sizes derived from the same study were rarely reported, the covariance among dependent effect sizes could not be directly estimated. Therefore, a diagonal variance structure based on the estimated sampling variances was used, while dependence among effect sizes was partially addressed through the nested random-effects structure. Nevertheless, potential correlations caused by shared-control designs could not be fully modeled and were further evaluated through sensitivity analyses. To evaluate the potential influence of including multiple effect sizes from the same study, additional one-comparison-per-study sensitivity analyses were conducted for all outcomes. The comparison with the largest total sample size was selected. When sample sizes were equal, the dose closest to the within-study median dose was retained, and remaining ties were resolved according to the original data-extraction order. The selected effect sizes were synthesized using conventional random-effects models. Model parameters and variance components were estimated using restricted maximum likelihood. Heterogeneity was summarized using the multilevel extension of the I^2^ statistic.

#### 2.5.2. Subgroup Analyses

To explore potential sources of between-study heterogeneity, predefined subgroup analyses were conducted according to study characteristics, and between-group differences were assessed. The main subgroup variables included: (1) physiological stage (weaned piglets, growing pigs, and finishing pigs); (2) flavonoid subclass (e.g., flavonols, isoflavones, and proanthocyanidins); and (3) sample source or measurement site (only for antioxidant outcomes), including specific biological samples (e.g., serum, plasma, liver, and jejunal mucosa) and grouped tissue categories (blood, intestinal tissue, and liver). Additional subgroup estimates are presented in Supplementary Table S7.

#### 2.5.3. Dose–Time Meta-Regression Analyses

Dose–time meta-regression analyses were restricted to comparisons for which supplementation doses could be reliably standardized to mg/kg diet and intervention-duration data were available. Analyses were conducted separately according to physiological stage. Model estimation was attempted for each stage, and models that could not be reliably estimated were reported as unavailable. Original and standardized doses are presented in Supplementary Table S11, and the numbers of effect sizes and independent studies included in each stage-specific analysis are reported in Supplementary Table S6. Hedges’ *g* was used as the effect size. Dose, scaled per 100 mg/kg diet, and intervention duration were entered simultaneously as continuous moderators in three-level random-effects models. Standardized dose represented the total supplemented product rather than analytically verified active flavonoid exposure and was therefore interpreted as supplementation intensity. For the successfully estimated models, dose–duration correlations were weak to moderate (r=−0.067 for weaned pigs and r=−0.427 for finishing pigs) and below a collinearity threshold of |r|=0.80. Model performance was evaluated using joint moderator tests and pseudo-$R^{2}$ values, with interpretation taking into account the number of independent studies. Although both linear and nonlinear dose–response meta-analytic approaches are available [21,22], only linear additive models were fitted because the available data did not support reliable estimation of nonlinear relationships.

#### 2.5.4. Publication Bias and Sensitivity Analyses

Egger’s linear regression test was used to assess potential small-study effects and funnel plot asymmetry [23], with p<0.05 considered indicative of funnel plot asymmetry. For outcomes showing potential publication bias, additional analyses were conducted using the original mean difference (MD) scale within the Egger framework to evaluate whether data standardization may have influenced the observed asymmetry. Sensitivity analyses were conducted using two complementary approaches. Because conventional influence diagnostics may be unstable for multilevel models with limited numbers of independent studies, the influence of individual studies was assessed using leave-one-study-out analyses and one-comparison-per-study sensitivity analyses. First, one-comparison-per-study sensitivity analyses were performed to evaluate the potential influence of dependent effect sizes. For each study, the comparison with the largest total sample size was retained, and the selected comparisons were synthesized using conventional random-effects models. Second, leave-one-study-out analyses were conducted based on the primary three-level models to evaluate whether removal of potentially influential individual studies substantially affected the pooled estimates. In addition, Rosenthal’s fail-safe N (FSN) was calculated as a supplementary robustness indicator [24].

#### 2.5.5. Risk of Bias and Certainty of Evidence Assessment

**Figure 2:**
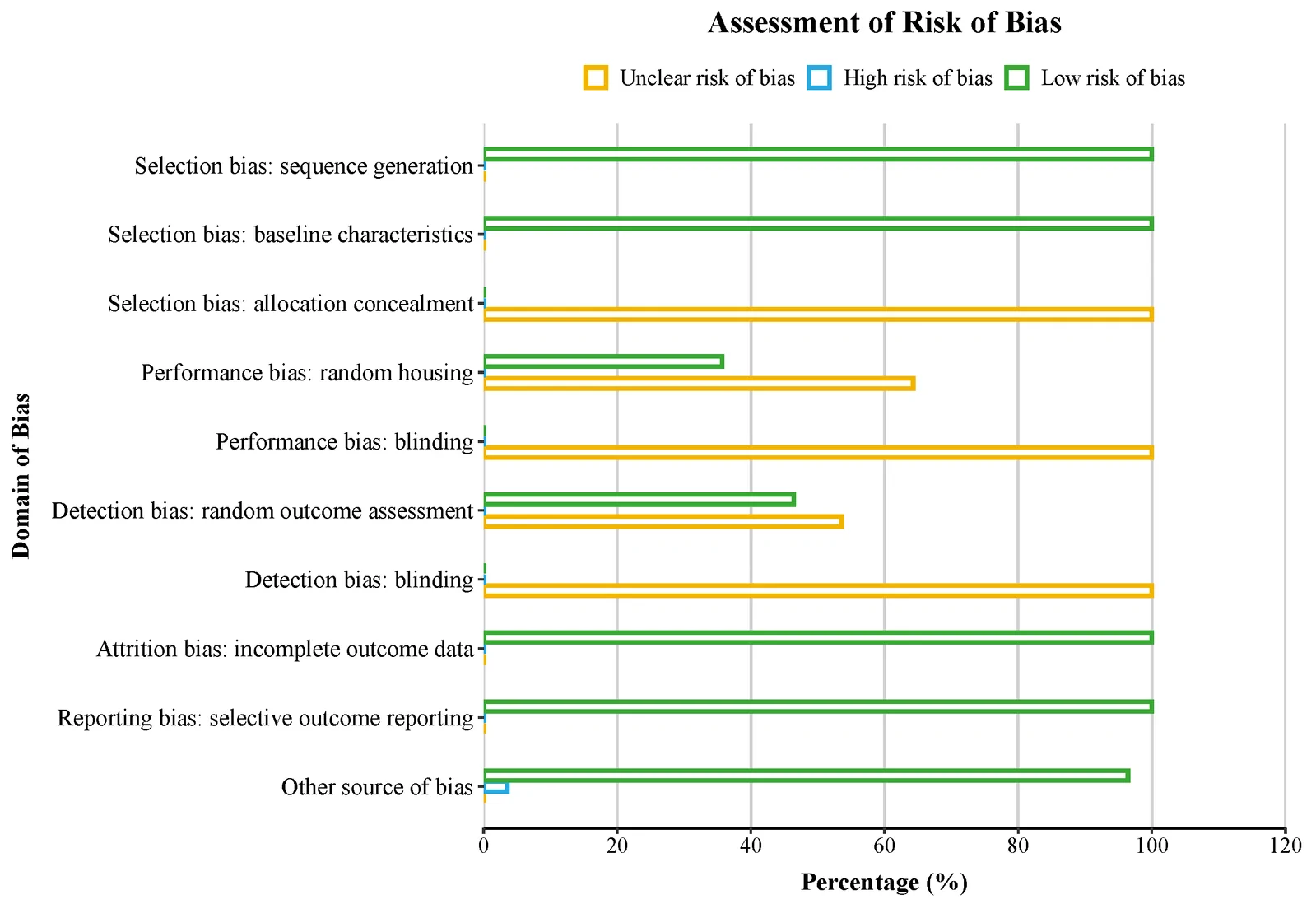
Risk of bias assessment of the included studies based on the SYRCLE risk of bias tool.

Two investigators independently evaluated the methodological quality of the included animal experiments using the Systematic Review Centre for Laboratory Animal Experimentation (SYRCLE) risk-of-bias tool [25]. The assessment covered ten domains related to selection, performance, detection, attrition, reporting, and other potential sources of bias. Each domain was classified as having a low, high, or unclear risk of bias. Disagreements were resolved through discussion, with arbitration by a third investigator when required. Unclear judgments were concentrated mainly in the allocation concealment and blinding domains because these procedures were insufficiently reported. The overall distribution of judgments is shown in Figure 2, with domain-level proportions and study-level judgments presented in Supplementary Tables S4 and S9, respectively.

The certainty of evidence for ADG, ADFI, F/G, SOD, MDA, and GSH-Px was evaluated using an adapted Grading of Recommendations Assessment, Development and Evaluation (GRADE) framework [26]. Because GRADE was originally developed primarily for clinical evidence, its domains were adapted to the characteristics of animal experimental research, with reference to methodological guidance on applying GRADE to preclinical animal evidence [27]. Evidence from randomized controlled trials was initially rated as high certainty and was downgraded when serious concerns were identified regarding risk of bias, inconsistency, indirectness, imprecision, or publication bias. Evidence certainty was rated as low for ADG and moderate for ADFI, F/G, SOD, MDA, and GSH-Px. The rationale for each downgrading decision is provided in Supplementary Table S3.

## 3. Results

### 3.1. Literature Search and Screening Results

A comprehensive search retrieved 7633 records from four databases. After 1115 duplicates were removed and 6371 irrelevant records were excluded during title and abstract screening, 147 reports were sought for retrieval. The full texts of 15 potentially eligible reports remained unavailable after retrieval attempts; therefore, their eligibility could not be assessed. The remaining 132 articles were assessed for eligibility according to the predefined criteria. After full-text review, 104 studies were excluded for the reasons shown in Figure 1. Ultimately, 28 randomized controlled trials (RCTs) were included in the meta-analysis. The characteristics of the included studies are presented in Table 1.

### 3.2. Associations of Flavonoid Supplementation with Growth Performance in Pigs

The overall pooled estimates for growth performance and antioxidant outcomes are summarized in Table 2.

#### 3.2.1. Average Daily Gain (ADG)

**Figure 3:**
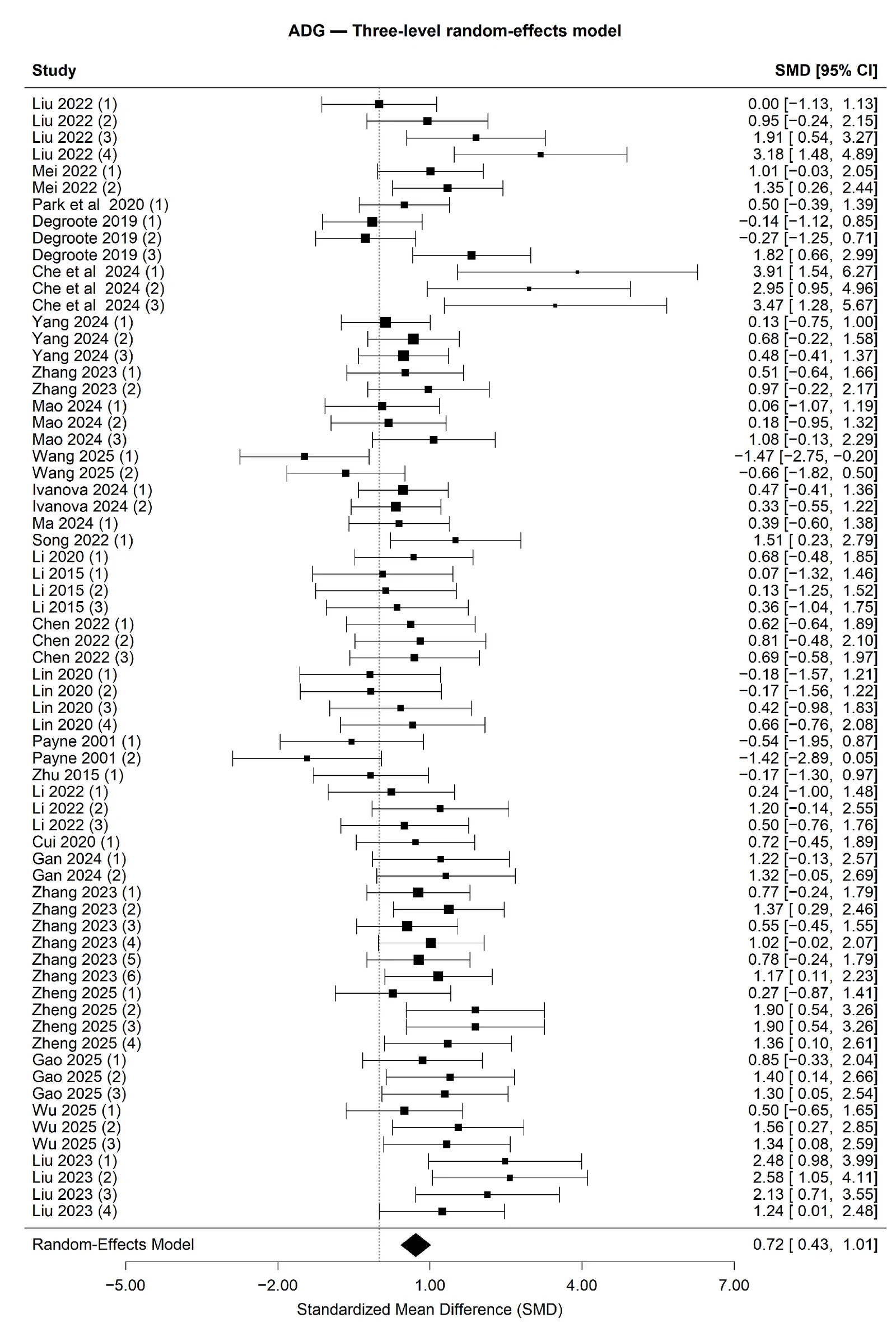
Forest plot of the effect of dietary flavonoids on average daily gain (ADG) in pigs. The included studies are cited in the figure: [28,29,30,31,32,33,34,35,36,37,38,39,40,41,42,43,44,45,46,47,48,49,50,51,52,53]. Square areas are proportional to the weights assigned to individual effect sizes in the three-level random-effects model.

The three-level meta-analysis indicated that flavonoid supplementation was associated with higher ADG in pigs (67 effect sizes from 26 studies; SMD = 0.719, 95% CI: 0.428 to 1.009; p<0.001). Heterogeneity was moderate ($I^{2}=51.85\%$), indicating that variability remained among studies after the model accounted for multiple comparisons within studies (Figure 3).

#### 3.2.2. Average Daily Feed Intake (ADFI)

**Figure 4:**
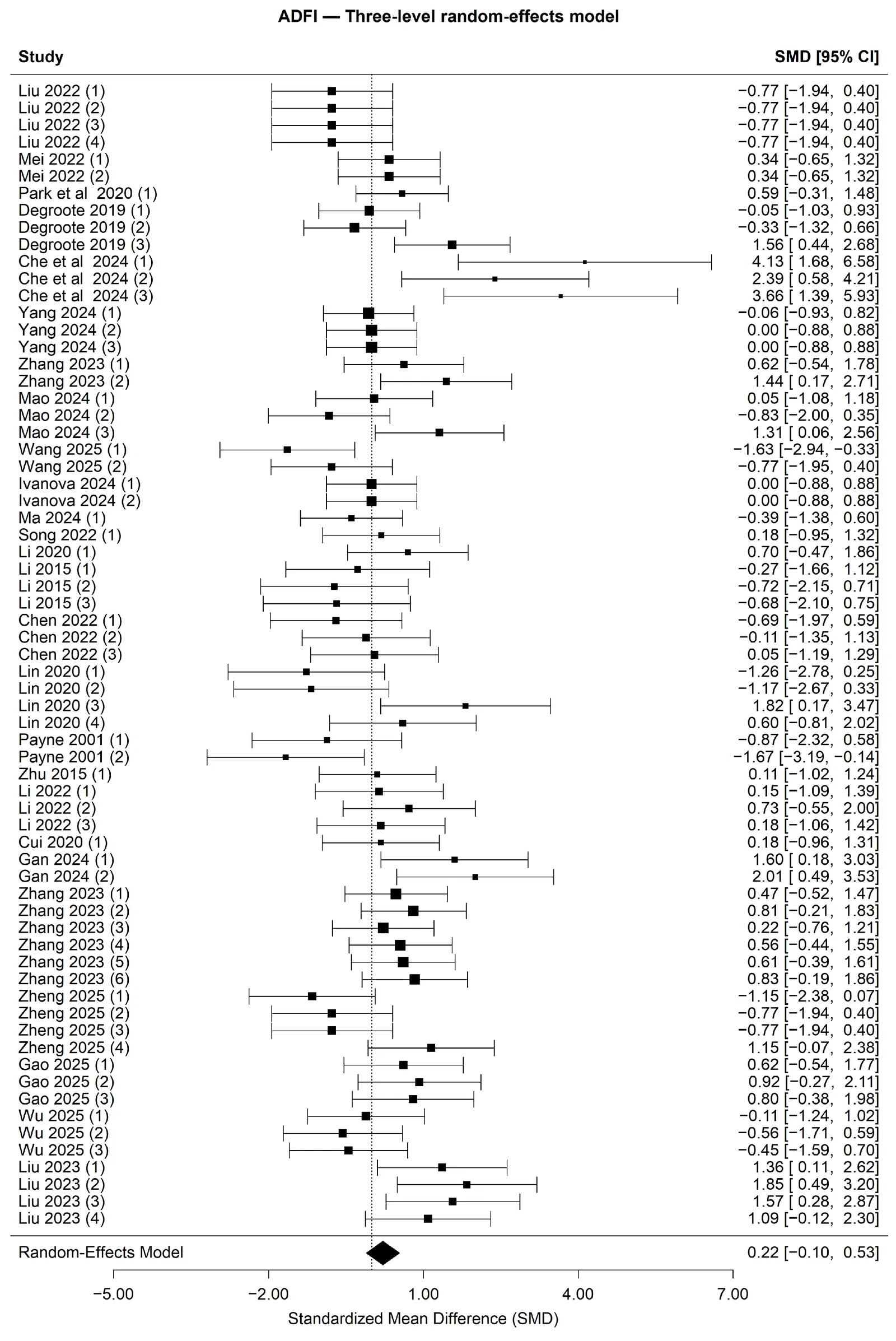
Forest plot of the effect of dietary flavonoids on average daily feed intake (ADFI) in pigs. The included studies are cited in the figure: [28,29,30,31,32,33,34,35,36,37,38,39,40,41,42,43,44,45,46,47,48,49,50,51,52,53]. Square areas are proportional to the weights assigned to individual effect sizes in the three-level random-effects model.

The three-level meta-analysis yielded a positive but non-significant estimate for average daily feed intake (ADFI) in pigs (67 effect sizes from 26 studies; SMD = 0.217, 95% CI: −0.101 to 0.535; p=0.182). Compared with analyses assuming independent effect sizes, the three-level model provided a more conservative estimate by accounting for the dependence of multiple comparisons within the same study. The estimated heterogeneity was moderate ($I^{2}=59.30\%$), indicating considerable variability among studies after accounting for within-study dependence (Figure 4). Subgroup analyses by physiological stage did not detect a statistically significant between-group difference for ADFI (p=0.447). The estimated effects were 0.350 in weaned pigs (46 effect sizes from 18 studies; 95% CI: −0.030 to 0.731; p=0.071), 0.028 in growing pigs (7 effect sizes from 3 studies; 95% CI: −0.348 to 0.404; p=0.884), and −0.150 in finishing pigs (14 effect sizes from 5 studies; 95% CI: −1.092 to 0.793; p=0.756). Thus, the final subgroup analysis did not provide evidence that flavonoid supplementation was associated with ADFI at any individual physiological stage.

#### 3.2.3. Feed-to-Gain Ratio (F/G)

**Figure 5:**
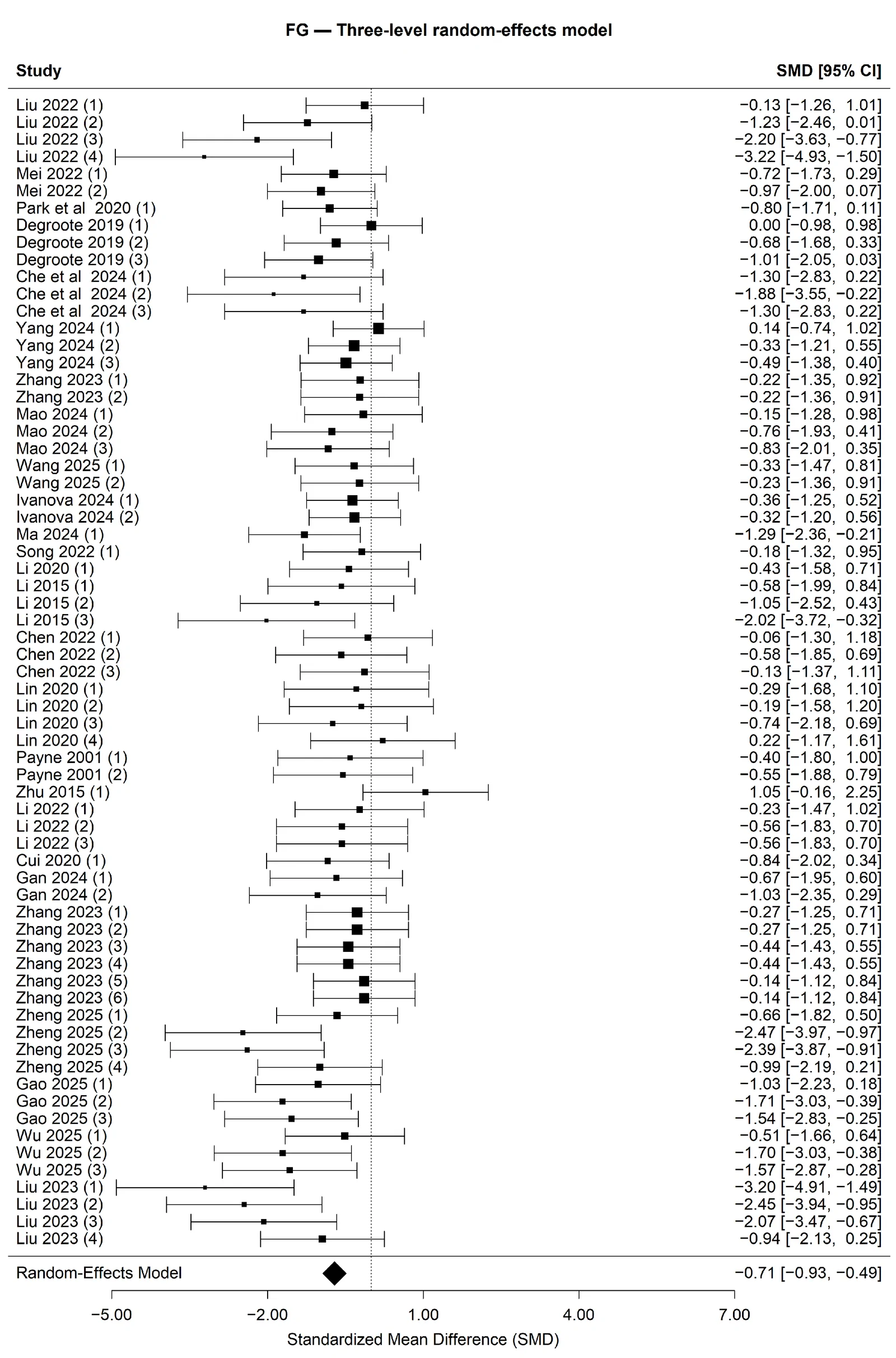
Forest plot of the effect of dietary flavonoids on feed-to-gain ratio (F/G) in pigs. The included studies are cited in the figure: [28,29,30,31,32,33,34,35,36,37,38,39,40,41,42,43,44,45,46,47,48,49,50,51,52,53]. Square areas are proportional to the weights assigned to individual effect sizes in the three-level random-effects model.

Flavonoid supplementation was associated with a lower feed-to-gain ratio (F/G) (67 effect sizes from 26 studies; SMD = −0.708, 95% CI: −0.929 to −0.487; p<0.001), with low-to-moderate heterogeneity ($I^{2}=32.25\%$; Figure 5). The direction and magnitude of the effect remained consistent after accounting for dependent effect sizes using the three-level random-effects model, indicating that flavonoid supplementation was associated with improved feed utilization efficiency. The class subgroup test indicated a statistically significant between-group difference for F/G (p=0.014). The estimated effects were −0.594 for flavonols (23 effect sizes from 10 studies; 95% CI: −0.847 to −0.341; p<0.001), −0.565 for isoflavones (23 effect sizes from 9 studies; 95% CI: −0.976 to −0.154; p=0.007), −0.844 for flavanones (3 effect sizes from 2 studies; 95% CI: −1.568 to −0.120; p=0.022), and −1.768 for proanthocyanidins (8 effect sizes from 2 studies; 95% CI: −2.406 to −1.130; p<0.001). However, the estimates for flavanones and proanthocyanidins should be interpreted cautiously because each was based on only two independent studies.

#### 3.2.4. Dose–Time Meta-Regression Results

**Figure 6:**
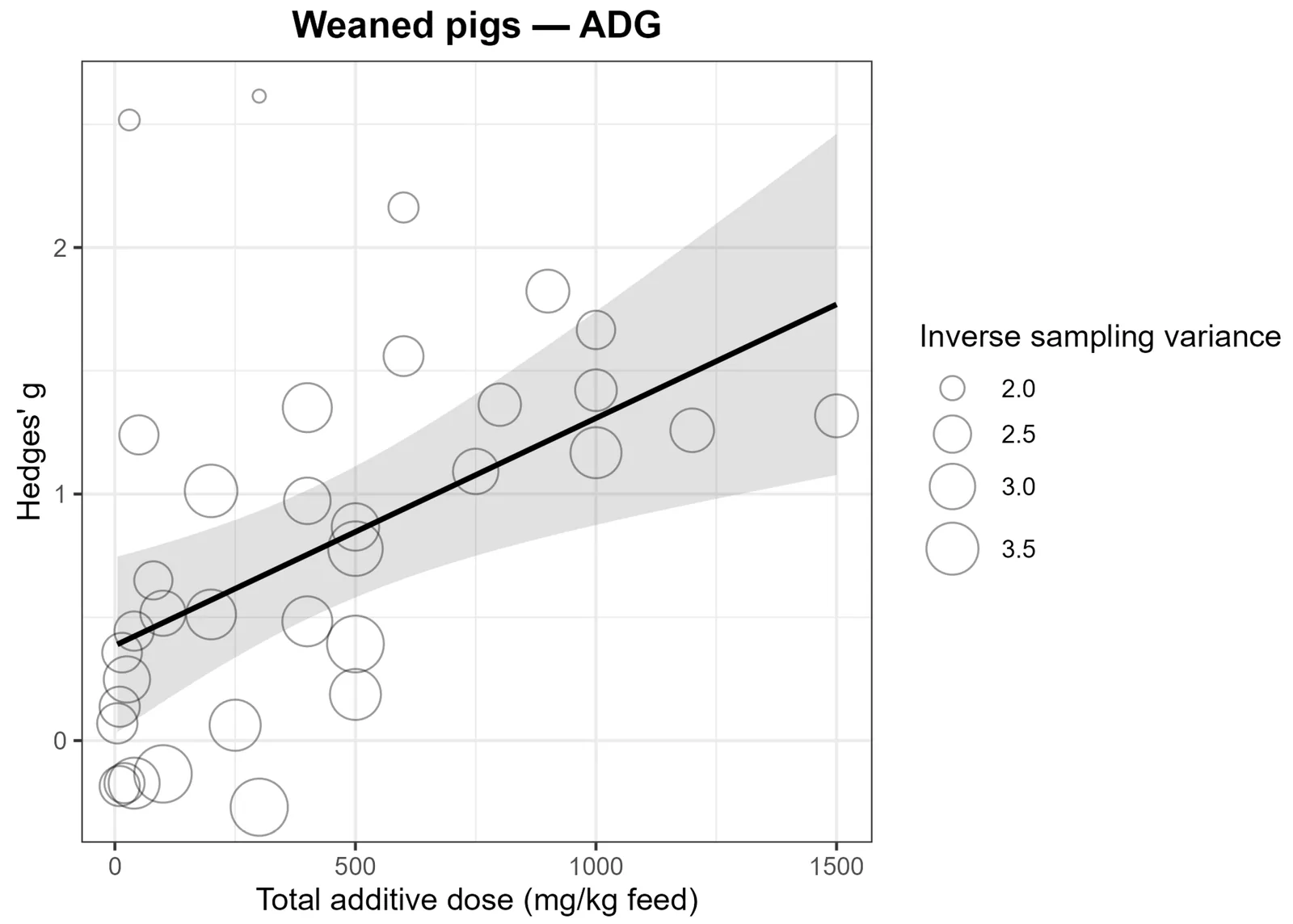
Bubble plot of the association between supplementation dose and average daily gain (ADG) in weaned pigs. Bubble size is proportional to inverse sampling variance, and the fitted line indicates the estimated linear association.

To further explore potential sources of heterogeneity, three-level meta-regression models incorporating supplementation dose and intervention duration were constructed using Hedges’ *g* as the effect size. These models accounted for the hierarchical dependence of multiple effect sizes nested within studies. Among weaned pigs, 35 effect sizes from 14 studies were included (Figure 6). Supplementation intensity showed a positive relationship with ADG (β=0.0922 per 100 mg/kg increase, 95% CI: 0.0332 to 0.1513; p=0.0022), whereas intervention duration was not associated with ADG (β=0.0016 per day, 95% CI: −0.0108 to 0.0141; p=0.797). The joint moderator test was statistically significant (p=0.009), and the model yielded a pseudo-$R^{2}$ of 49.88%. For F/G in weaned pigs, neither supplementation dose (β=−0.0299, 95% CI: −0.0963 to 0.0364; p=0.376) nor intervention duration (β=−0.0041 per day, 95% CI: −0.0214 to 0.0131; p=0.637) was statistically significant. The joint moderator test was also non-significant (p=0.615), and the pseudo-$R^{2}$ was 0%. The models for growing pigs could not be reliably estimated because only seven effect sizes from three independent studies were available. Among finishing pigs, 12 effect sizes from four studies were included. Supplementation dose (β=0.1662 per 100 mg/kg increase, 95% CI: 0.0630 to 0.2694; p=0.0016) and intervention duration (β=0.0170 per day, 95% CI: 0.0016 to 0.0324; p=0.031) were positively associated with ADG. The joint moderator test was statistically significant (p=0.004), and the pseudo-$R^{2}$ was approximately 100%. For F/G in finishing pigs, supplementation dose (β=−0.1634, 95% CI: −0.2670 to −0.0598; p=0.0020) and intervention duration (β=−0.0229 per day, 95% CI: −0.0384 to −0.0074; p=0.0037) were associated with lower F/G values. The joint moderator test was statistically significant (p=0.002), and the pseudo-$R^{2}$ was approximately 100%. These finishing-pig findings should be regarded as exploratory because only four independent studies contributed to the models. The high pseudo-$R^{2}$ values may reflect model overfitting, and the independent contribution of intervention duration could not be separated from those of other related study characteristics.

Overall, these findings suggest that supplementation intensity and intervention duration may be associated with growth performance outcomes at specific production stages. However, because the included studies differed in flavonoid sources, chemical composition, extraction procedures, and bioavailability, the observed dose-related patterns should be interpreted as statistical associations rather than precise quantitative dose requirements.

### 3.3. Associations of Flavonoid Supplementation with Antioxidant Capacity in Pigs

#### 3.3.1. Superoxide Dismutase (SOD)

**Figure 7:**
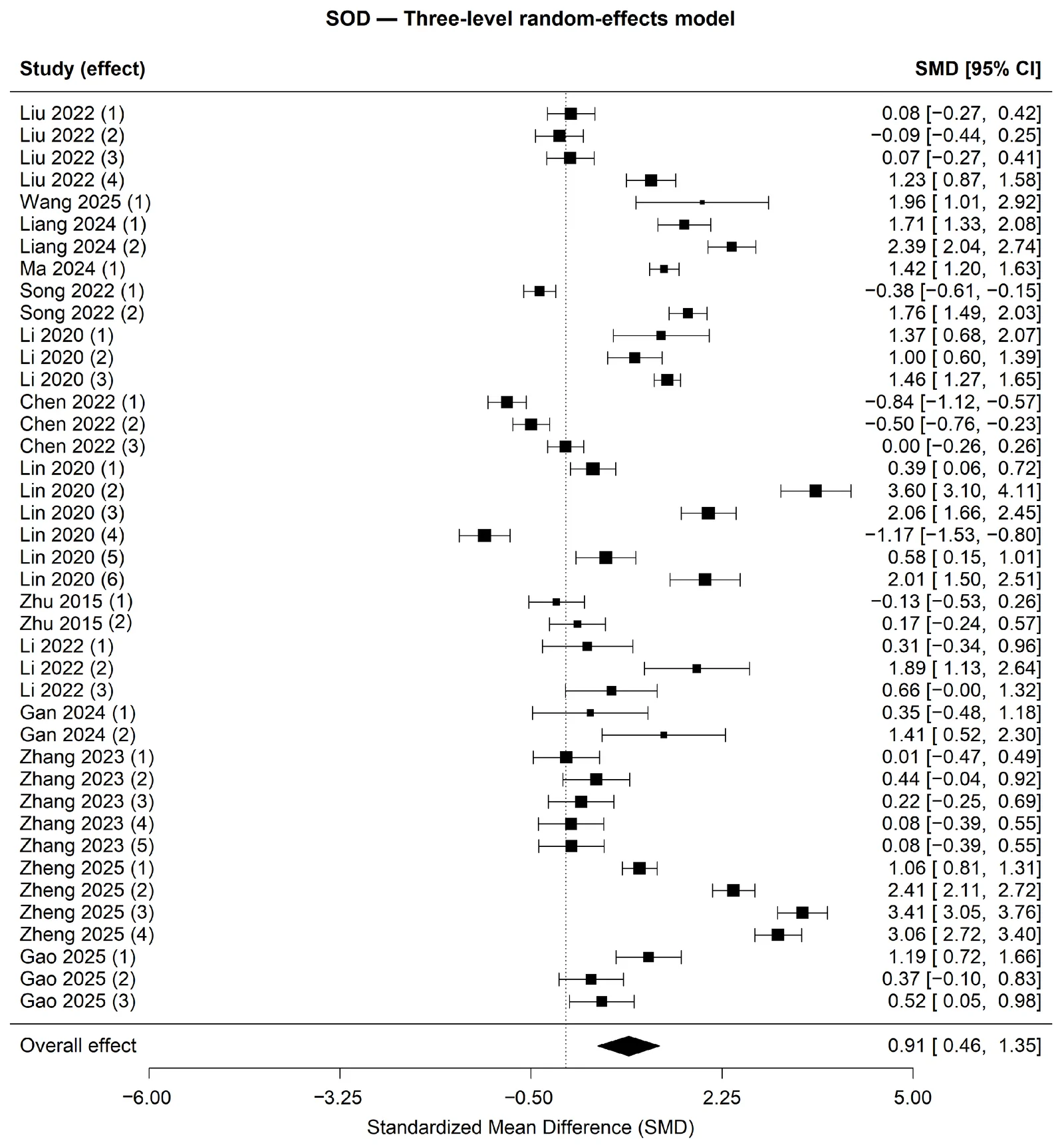
Forest plot of the effect of dietary flavonoids on superoxide dismutase (SOD) activity in pigs. The included studies are cited in the figure: [28,36,38,39,40,42,43,45,46,48,49,50,51,54]. Square areas are proportional to the weights assigned to individual effect sizes in the three-level random-effects model.

Flavonoid supplementation was associated with increased SOD activity in pigs (41 effect sizes from 14 studies; SMD = 0.907, 95% CI: 0.462 to 1.351; p<0.001; Figure 7). However, substantial heterogeneity remained after accounting for dependent effect sizes ($I^{2}=97.29\%$), indicating considerable variability among studies and experimental conditions.

Subgroup analyses by tissue group did not detect a statistically significant between-group difference (p=0.313). The pooled estimates were 0.774 for blood (34 effect sizes from 14 studies), 1.696 for intestinal tissue (4 effect sizes from 4 studies), and 1.286 for liver (3 effect sizes from 3 studies). These subgroup estimates should be interpreted cautiously because of the limited number of contributing studies and the substantial heterogeneity observed across comparisons.

Although larger estimated effects were observed in some intestinal measurements, these findings should be considered exploratory rather than evidence of a tissue-specific mechanism. Differences in study design, sample characteristics, and measurement methods may contribute to the observed variation.

The class subgroup test was not statistically significant (p=0.281). The estimated SOD effects were 1.068 for flavonols (6 effect sizes from 3 studies) and 0.683 for isoflavones (17 effect sizes from 6 studies). Thus, the available analysis does not establish a difference between flavonoid classes.

Subgroup analyses by physiological stage did not detect a statistically significant between-group difference for SOD (p=0.146). The estimates were 1.244 in finishing pigs (10 effect sizes from 3 studies; 95% CI: −0.089 to 2.576; p=0.067) and 0.921 in weaned pigs (28 effect sizes from 11 studies; 95% CI: 0.530 to 1.311; p<0.001).

#### 3.3.2. Malondialdehyde (MDA)

**Figure 8:**
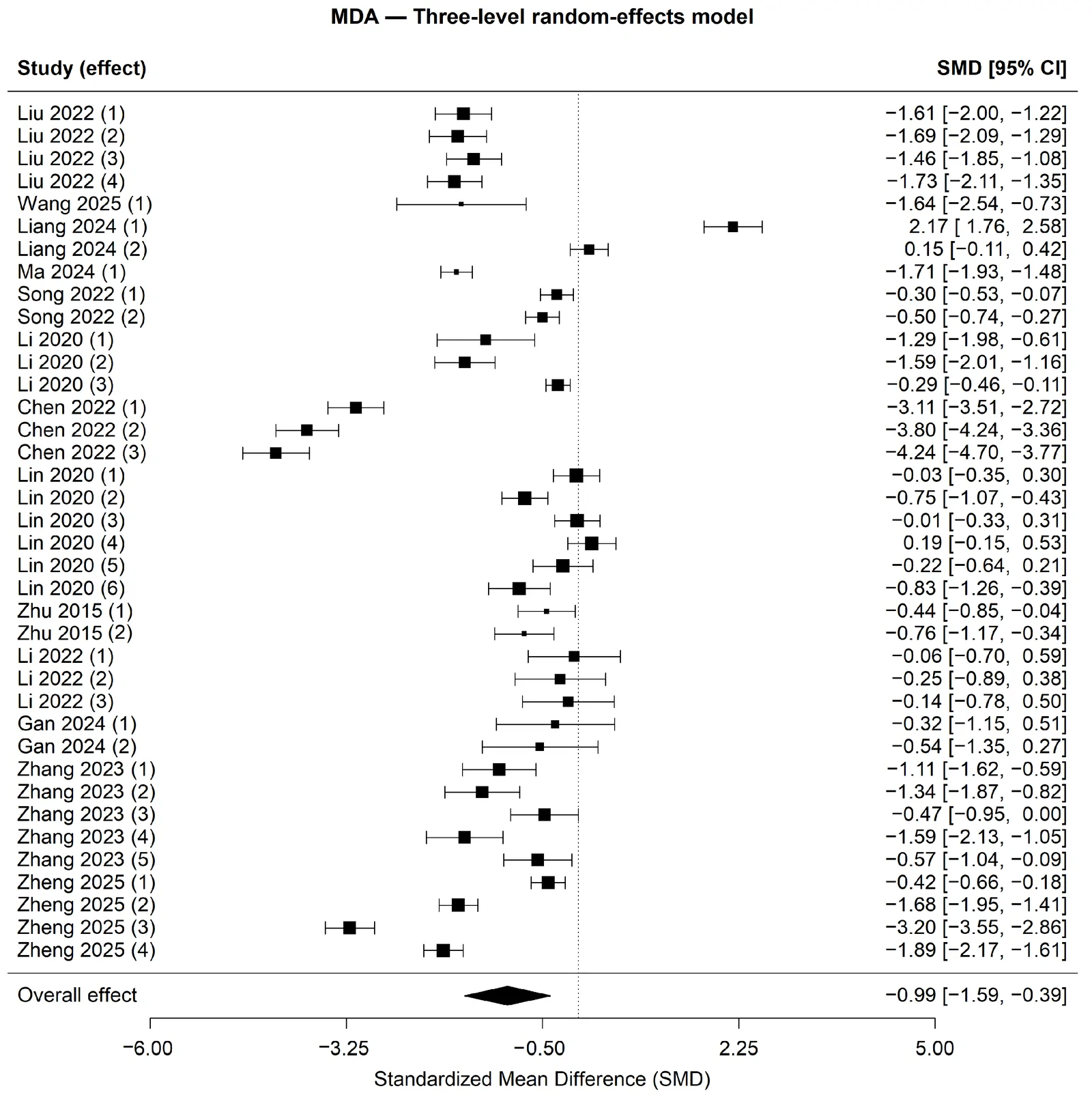
Forest plot of the effect of dietary flavonoids on malondialdehyde (MDA) levels in pigs. The included studies are cited in the figure: [28,36,38,39,40,42,43,45,46,48,49,50,54]. Square areas are proportional to the weights assigned to individual effect sizes in the three-level random-effects model.

The three-level meta-analysis showed that dietary flavonoid supplementation was associated with lower MDA concentrations in pigs (38 effect sizes from 13 studies; SMD = −0.989, 95% CI: −1.592 to −0.386; p=0.001; Figure 8). However, substantial heterogeneity remained after accounting for dependent effect sizes ($I^{2}=97.83\%$), suggesting considerable variability among studies, experimental conditions, and outcome measurements.

The class subgroup test was not statistically significant (p=0.548). The estimated MDA effects were −1.658 for flavonols (6 effect sizes from 3 studies) and −1.063 for isoflavones (17 effect sizes from 6 studies), with the confidence interval for isoflavones crossing zero.

Subgroup analyses by physiological stage detected a statistically significant between-group difference for MDA (p<0.001). The estimated reduction was larger in finishing pigs (10 effect sizes from 3 studies; SMD = −1.426, 95% CI: −2.089 to −0.763; p<0.001) than in weaned pigs (25 effect sizes from 10 studies; SMD = −0.564, 95% CI: −1.051 to −0.077; p=0.023). However, this finding should be interpreted cautiously because the finishing-pig estimate was based on only three independent studies.

Subgroup analyses by tissue group did not detect a statistically significant between-group difference (p=0.065). The estimated effects were −0.884 for blood (31 effect sizes from 13 studies), −0.989 for intestinal tissue (4 effect sizes from 4 studies), and −0.350 for liver (3 effect sizes from 3 studies). These estimates should be considered exploratory because some groups contained few studies.

Overall, the stage subgroup test suggested a difference in MDA responses between finishing and weaned pigs, whereas the class, sample-type, and tissue-group tests were not statistically significant. Because heterogeneity remained substantial and several subgroup estimates were based on few studies, these findings should be interpreted cautiously.

#### 3.3.3. Glutathione Peroxidase (GSH-Px)

**Figure 9:**
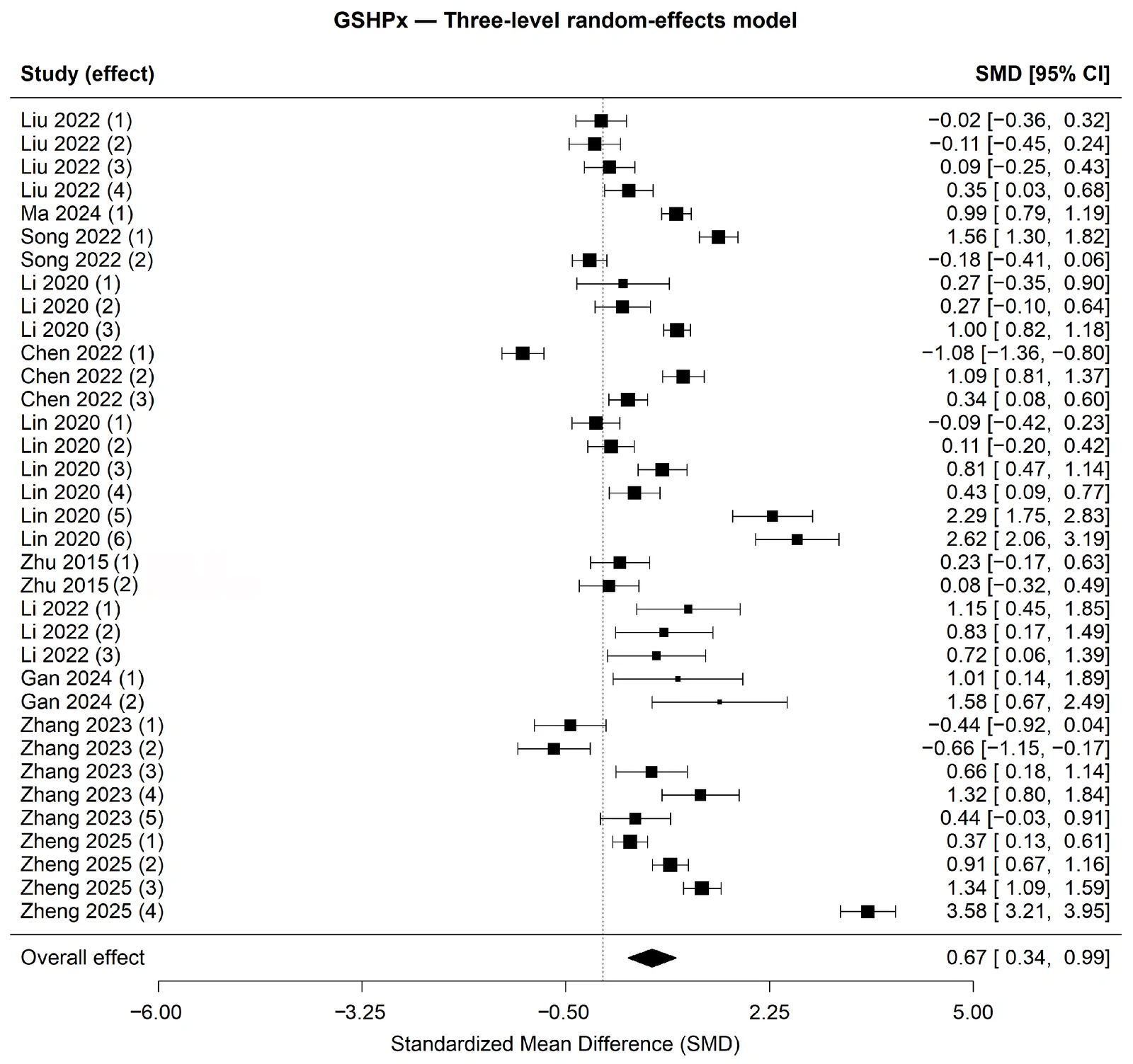
Forest plot of the effect of dietary flavonoids on glutathione peroxidase (GSH-Px) activity in pigs. The included studies are cited in the figure: [28,38,39,40,42,43,45,46,48,49,50]. Square areas are proportional to the weights assigned to individual effect sizes in the three-level random-effects model.

The three-level meta-analysis showed that flavonoid supplementation increased GSH-Px activity in pigs (35 effect sizes from 11 studies; SMD = 0.666, 95% CI: 0.339 to 0.994; p<0.001; Figure 9). Substantial heterogeneity was observed among studies ($I^{2}=96.47\%$), indicating considerable variability in antioxidant responses across experimental conditions. Subgroup analyses by tissue group did not detect a statistically significant between-group difference (p=0.348). The estimated effects were 0.585 for blood (29 effect sizes from 11 studies), 1.278 for intestinal tissue (3 effect sizes from 3 studies), and 1.021 for liver (3 effect sizes from 3 studies). However, the tissue-specific estimates should be interpreted cautiously because they were derived from limited numbers of studies and substantial heterogeneity remained within subgroups. Subgroup analyses by physiological stage did not detect a statistically significant between-group difference for GSH-Px (p=0.476). The estimates were 0.933 in finishing pigs (10 effect sizes from 3 studies; 95% CI: −0.037 to 1.903; p=0.059) and 0.646 in weaned pigs (22 effect sizes from 8 studies; 95% CI: 0.313 to 0.979; p<0.001). Overall, the pooled analysis supports an increase in GSH-Px activity, whereas the subgroup findings do not confirm a tissue-specific response pattern. These findings should be interpreted in the context of the substantial heterogeneity among studies.

### 3.4. Sensitivity Analyses and Publication Bias Assessment

The one-comparison-per-study sensitivity analyses yielded estimates of 0.665 for ADG, 0.074 for ADFI, −0.707 for F/G, 1.301 for SOD, −1.159 for MDA, and 0.721 for GSH-Px. The direction and statistical significance of all outcomes were consistent with the primary three-level models, supporting the qualitative robustness of the findings, although substantial heterogeneity remained for the antioxidant outcomes (Supplementary Table S10).

Egger’s tests on the SMD scale indicated funnel plot asymmetry for ADG (P=0.030) and F/G (p=0.0068). On the original MD scale, the result was no longer significant for ADG (p=0.447) but remained significant for F/G (p=0.010). Trim-and-fill analyses produced statistically significant adjusted estimates for both ADG (SMD = 0.441) and F/G (SMD = −0.599). The fail-safe *N* values were 297 for ADG, 366 for F/G, and 0 for ADFI. Complete diagnostics and funnel plots are provided in Supplementary Table S5 and Figures S1 and S6, respectively.

## 4. Discussion

### 4.1. Potential Biological Explanations for Growth Responses to Dietary Flavonoids

Beyond quantifying the overall associations of dietary flavonoids with swine growth, our dose–time meta-regression explored whether supplementation intensity and intervention duration were associated with growth-related outcomes at different physiological stages. The pooled analysis indicated higher ADG and lower F/G, whereas the overall association with ADFI was not statistically significant. Subgroup tests did not detect significant differences among physiological stages for ADG (p=0.889), ADFI (p=0.447), or F/G (p=0.406). Accordingly, the available evidence does not establish distinct stage-specific response patterns for these outcomes. During the weaning transition, previous studies have suggested that flavonoids may help alleviate stress-associated physiological disturbances. Potential mechanisms include regulation of local inflammatory responses [56,57,58] and maintenance of intestinal barrier integrity to support nutrient absorption [59,60]. These biological effects may contribute to the growth-related associations reported in previous studies [61]; however, they were not directly evaluated in the present meta-analysis and do not explain a confirmed difference among physiological stages. Conversely, the dose–time meta-regression suggested that intervention duration was statistically associated with growth responses in finishing pigs. However, because the number of independent studies in this subgroup was limited, this observation should be interpreted cautiously and does not establish a causal effect of intervention duration. It should also be noted that intervention duration was not independent of other study characteristics. Differences in supplementation duration may be associated with variations in animal age, physiological stage, flavonoid sources, dosage levels, health status, and basal diet composition. Therefore, the observed associations between intervention duration and growth responses should not be interpreted as an isolated causal effect of supplementation duration. Without the acute stress of weaning, previous studies have suggested that long-term flavonoid supplementation may influence intestinal microbial composition and inflammatory regulation. However, these mechanisms were not directly evaluated in the included studies and should therefore be considered biological hypotheses rather than conclusions derived from the present meta-analysis [62].

Previous studies have also proposed that reduced inflammatory burden may influence nutrient allocation and energy metabolism [63,64]. However, inflammatory markers and metabolic pathways were not directly assessed in the present meta-analysis. While differences in experimental duration between weaning and finishing studies may have contributed to the temporal patterns observed in the meta-regression, the potential role of supplementation duration requires confirmation from additional standardized studies. From a quantitative perspective, the pooled effects indicate that flavonoid supplementation was associated with statistically significant improvements in growth performance, with moderate effect sizes for ADG and F/G (SMD = 0.719 and SMD = −0.708, respectively). These findings suggest a consistent overall direction of growth responses across included studies. However, the magnitude of these effects should be interpreted in light of the heterogeneity among studies (ADG: $I^{2}=51.85\%$; F/G: $I^{2}=32.25\%$) and differences in flavonoid preparations, supplementation strategies, and experimental conditions. Therefore, the practical magnitude of benefits may vary among production systems.

### 4.2. Associations of Dietary Flavonoids with Systemic and Tissue Antioxidant Capacity

Dietary flavonoid supplementation was associated with beneficial changes in antioxidant indicators in pigs, including increased SOD and GSH-Px activities and reduced MDA concentrations. Nevertheless, the magnitude of these responses varied among studies, suggesting that these associations may vary according to experimental settings and physiological status.

Subgroup analyses indicated that some intestinal measurements had numerically larger effect estimates than blood measurements. For SOD, the estimate was 1.696 for intestinal tissue (4 effect sizes from 4 studies) and 0.774 for blood (34 effect sizes from 14 studies). However, these differences may reflect variation in baseline physiological status, sample size, and measurement methods rather than indicating a stronger response in a specific tissue.

The intestine is an important interface between dietary components and host physiological responses, and intestinal epithelial cells are continuously exposed to dietary oxidants and microbial challenges [65]. Previous studies have suggested that flavonoids may influence oxidative stress through direct radical-scavenging activity [66] and antioxidant-related pathways, including Nrf2 signaling [67,68]. However, these mechanisms were not directly evaluated in the studies included in this meta-analysis and should be regarded as potential biological explanations rather than conclusions derived from the pooled estimates.

Although relatively large effect estimates were observed in some mucosal subgroups, these findings should be regarded as exploratory because they were based on few comparisons. Variation in baseline oxidative status, metabolic characteristics, experimental conditions, and other biological factors may contribute to differences in antioxidant responses among studies [69,70]. Further standardized studies are needed to determine whether responses to flavonoid supplementation consistently differ among tissues. The substantial heterogeneity in antioxidant outcomes may reflect differences in flavonoid subclass and bioavailability, the use of purified compounds or plant-derived extracts, the tissues sampled, and other experimental conditions. Variation in physiological stage, basal diet, health status, and supplementation strategy may also contribute to residual heterogeneity. Overall, flavonoid supplementation was associated with increased SOD and GSH-Px activities and reduced MDA concentrations. However, substantial between-study heterogeneity and the moderate certainty of evidence indicate that these effects may vary across flavonoid preparations and experimental conditions. The pooled estimates should therefore be interpreted as evidence of potential antioxidant benefits rather than uniform responses to flavonoid supplementation.

### 4.3. Structure–Activity Differences and Functional Characteristics of Flavonoid Subclasses

The between-class subgroup test was statistically significant for F/G (P=0.014) but not for ADG (P=0.388), ADFI (P=0.826), SOD (P=0.281), MDA (P=0.548), or GSH-Px (P=0.234). Proanthocyanidins showed numerically larger estimated effects in the subgroup analyses for some growth-related outcomes, whereas flavonols showed a relatively larger estimated effect for MDA reduction. However, these subgroup findings should be interpreted cautiously because they were derived from heterogeneous flavonoid preparations and limited numbers of studies. The relatively larger antioxidant-related effects observed for flavonols, especially quercetin, have been associated with structural characteristics such as the C_3_-OH group in the C-ring and the catechol structure in the B-ring, which may influence electron donation and metal chelation [71]. In contrast, structures lacking the C_3_-OH group or with a saturated C-ring may show weaker radical-scavenging activity [72]. In the reported class analysis, both flavonols and isoflavones were associated with increased SOD activity, but the between-class test was not statistically significant (P=0.281); the present evidence therefore does not support a definitive class ranking.

For growth-related outcomes, proanthocyanidins had relatively large effect estimates for ADG (SMD = 1.641, 95% CI: 0.902 to 2.380; p<0.001) and F/G (SMD = −1.768, 95% CI: −2.406 to −1.130; p<0.001). However, each estimate was based on only two independent studies and should therefore be interpreted cautiously. Previous studies have suggested that these associations may involve changes in intestinal conditions and microbial composition [73]; however, these mechanisms were not directly evaluated in the present meta-analysis. Excessive intake may nevertheless impair nutrient utilization through protein precipitation and enzyme inhibition, reflecting the potential antinutritional properties of proanthocyanidins [74].

### 4.4. Limitations of the Present Study

Several limitations should be considered. First, methodological reporting and the certainty of the evidence should be interpreted cautiously. The SYRCLE assessment rated several domains as having an unclear risk of bias because procedures such as allocation concealment and blinding were insufficiently reported. Although unclear risk-of-bias judgments do not necessarily indicate confirmed methodological deficiencies, inadequate reporting limits the assessment of internal validity and may reduce confidence in the synthesized evidence. In addition, the adapted GRADE ratings represent assessments of confidence in the evidence within the context of animal nutrition research and are not directly equivalent to ratings used for clinical evidence. Future animal nutrition studies should report randomization, allocation concealment, and blinding procedures more transparently.

Second, residual dependence among effect sizes cannot be completely ruled out. Although three-level random-effects models and one-comparison-per-study sensitivity analyses were used to address multiple comparisons from the same study, the original studies rarely reported covariance information. This limitation prevented precise modeling of within-study correlations.

Third, the dose-related findings should be interpreted cautiously because the reported supplementation doses represented the total amounts of flavonoid-containing products rather than the actual amounts of active flavonoids. Differences in composition and bioavailability among purified compounds, plant-derived extracts, and commercial preparations may have contributed to variation in the observed responses.

Fourth, potential publication bias, residual heterogeneity, and limitations of the meta-regression analyses should be considered. Although the trim-and-fill and sensitivity analyses indicated that the main findings were qualitatively robust, funnel plot asymmetry may also reflect differences in flavonoid sources, animal stages, supplementation intensities, and experimental conditions. Few independent studies contributed to some stage-specific analyses, and the models for growing pigs could not be reliably estimated. Only linear additive models were fitted; therefore, the present findings do not exclude the possibility of nonlinear dose-related relationships. Furthermore, intervention duration was not experimentally manipulated, and its independent contribution could not be separated from other study characteristics.

Fifth, the full texts of 15 potentially eligible reports remained unavailable after retrieval attempts, preventing assessment of their eligibility. Their omission may have introduced selection bias.

## 5. Conclusions

This systematic review and meta-analysis suggests that dietary flavonoid supplementation may improve selected growth performance and antioxidant outcomes in pigs. Most subgroup tests did not detect statistically significant differences across physiological stages, flavonoid classes, sample types, or tissue groups; the exceptions were the class subgroup test for F/G and the stage subgroup test for MDA. These findings remain exploratory because some subgroup estimates were based on few independent studies and substantial heterogeneity remained for the antioxidant outcomes. The dose–time analyses identified potential associations of supplementation intensity and intervention duration with growth responses at specific physiological stages. However, these results do not establish optimal doses or durations because flavonoid source, active-compound content, experimental conditions, and study design varied among studies. Overall, the evidence supports potential nutritional benefits of dietary flavonoids in pigs but remains insufficient for specific practical recommendations. Additional well-designed studies using standardized and chemically characterized flavonoid preparations are needed to confirm these effects and develop evidence-based supplementation strategies. 

## Figures and Tables

**Table 1:** Characteristics of the studies included in the meta-analysis of dietary flavonoids in pigs.

Author (Year)	Stage	Source	Reported Dose	Outcomes Analyzed
Liu (2022) [28]	Finishing	Mulberry	200, 400, 800, 1600	ADG, ADFI, F/G, SOD, MDA, GSH-Px
Mei (2022) [29]	Weaned	Quercetin	200, 400	ADG, ADFI, F/G
Park (2020) [30]	Growing	Quercetin	0.1%	ADG, ADFI, F/G
Degroote (2019) [31]	Weaned	Quercetin	100, 300, 900	ADG, ADFI, F/G, MDA
Che (2024) [32]	Weaned	Astragalus	60, 30 mg/d	ADG, ADFI, F/G
Yang (2024) [33]	Growing	Quercetin	50, 150, 450	ADG, ADFI, F/G
Zhang (2023) [34]	Weaned	Quercetin	200, 400	ADG, ADFI, F/G, SOD, MDA, GSH-Px
Mao (2024) [35]	Weaned	Quercetin	250, 500, 750	ADG, ADFI, F/G
Wang (2025) [36]	Weaned	DHQ	100 (*P. copri*)	ADG, ADFI, F/G, SOD, MDA
Ivanova (2024) [37]	Finishing	DHQ	100, 200	ADG, ADFI, F/G
Ma (2024) [38]	Weaned	Rutin	500	ADG, ADFI, F/G, SOD, MDA, GSH-Px
Song (2022) [39]	Weaned	Mulberry	1000 g/t	ADG, ADFI, F/G, SOD, MDA, GSH-Px
Li (2020) [40]	Weaned	Soybean	C-SPC + ISF	ADG, ADFI, F/G, SOD, MDA, GSH-Px
Li (2015) [41]	Weaned	SI	5, 10, 15	ADG, ADFI, F/G
Chen (2022) [42]	Growing	Soybean	10, 20, 40	ADG, ADFI, F/G, SOD, MDA, GSH-Px
Lin (2020) [43]	Weaned	Soybean	10, 20, 40, 80	ADG, ADFI, F/G, SOD, MDA, GSH-Px
Payne (2001) [44]	Finishing	Soybean	2×, 5× ISF	ADG, ADFI, F/G
Zhu (2015) [45]	Weaned	ISO	40	ADG, ADFI, F/G, SOD, MDA, GSH-Px
Li (2022) [46]	Weaned	Daidzein	25, 50, 100	ADG, ADFI, F/G, SOD, MDA, GSH-Px
Cui (2020) [47]	Weaned	Citrus	300 mL/t	ADG, ADFI, F/G
Gan (2024) [48]	Finishing	F. aurantii	500, 1000	ADG, ADFI, F/G, SOD, MDA, GSH-Px
Zhang (2023) [49]	Weaned	Silymarin	0.05%, 0.1%	ADG, ADFI, F/G, SOD, MDA, GSH-Px
Zheng (2025) [50]	Finishing	Grape seed	15, 30, 60, 120	ADG, ADFI, F/G, SOD, MDA, GSH-Px
Gao (2025) [51]	Weaned	Baicalin	0.05, 0.1, 0.15%	ADG, ADFI, F/G, SOD
Wu (2025) [52]	Weaned	Pueraria	400, 600, 800	ADG, ADFI, F/G
Liu (2023) [53]	Weaned	Proantho.	30, 300–1200	ADG, ADFI, F/G
Liang (2024) [54]	Weaned	Luteolin	5000	SOD, MDA
Zou (2016) [55]	Finishing	Quercetin	25	MDA

**Notes:** Units are mg/kg unless otherwise specified. Reported doses are presented as originally described; doses used for meta-regression were harmonized to mg/kg diet where feasible. Standardized doses represent total additive supplementation intensity rather than absolute flavonoid content. Proantho., proanthocyanidins; DHQ, dihydroquercetin; ISF/ISO/SI, isoflavones; ADG, average daily gain; ADFI, average daily feed intake; F/G, feed-to-gain ratio; SOD, superoxide dismutase; MDA, malondialdehyde; GSH-Px, glutathione peroxidase.

**Table 2:** Overall meta-analysis of the effects of dietary flavonoids on growth performance and antioxidant capacity in pigs.

Outcome	*k*	SMD (95% CI)	*p*-Value	*I*^2^(%)
**Growth performance**				
ADG	67	0.719 [0.428, 1.009]	<0.001	51.85
ADFI	67	0.217 [−0.101, 0.535]	0.182	59.30
F/G	67	−0.708 [−0.929, −0.487]	<0.001	32.25
**Antioxidant capacity**				
SOD	41	0.907 [0.462, 1.351]	<0.001	97.29
MDA	38	−0.989 [−1.592, −0.386]	0.001	97.83
GSH-Px	35	0.666 [0.339, 0.994]	<0.001	96.47

Abbreviations: SMD, standardized mean difference; CI, confidence interval; ADG, average daily gain; ADFI, average daily feed intake; F/G, feed-to-gain ratio; SOD, superoxide dismutase; MDA, malondialdehyde; GSH-Px, glutathione peroxidase. Here, *k* represents the number of effect sizes included in each meta-analysis. Effect sizes were synthesized using three-level random-effects models to account for the hierarchical structure of effect sizes and partially address within-study dependence. Note: Bold text indicates outcome categories.

## Data Availability

The datasets analyzed in this study are provided in the Supplementary Materials and are available from the corresponding authors upon reasonable request.

## Supplementary Materials

The following supporting information can be downloaded at: https://www.mdpi.com/article/10.3390/antiox15080980/s1, Table S1: PRISMA 2020 Checklist and Location in Manuscript; Table S2: Detailed Search Strategy Used for Each Database; Table S3: GRADE Certainty Assessment and Reasons for Downgrading; Table S4: Summary of Risk of Bias Assessment Using the SYRCLE Tool; Table S5: Publication-Bias Diagnostics and Trim-and-Fill Analyses Based on One Comparison per Study; Table S6: Stage-Specific Three-Level Meta-Regression of Total Additive Dose and Intervention Duration for growth performance outcomes; Table S7: Complete Subgroup Analysis Results for Growth-Performance and Antioxidant Outcomes; Table S8: Leave-One-Study-Out Sensitivity Analysis Based on Three-Level Models; Table S9: Detailed Risk of Bias Judgments for Individual Included Studies Based on the SYRCLE Tool; Table S10: Sensitivity Analysis Using One Comparison per Study to Evaluate Dependent Effect Sizes; Table S11: Original Reported Doses and Standardized Supplementation Doses Used in the Dose–Time Meta-Regression Analyses; Figure S1: Funnel plots for publication-bias assessment based on datasets retaining one comparison per study; Figure S2: Leave-one-out sensitivity analyses for growth performance outcomes (ADG, ADFI, and F/G); Figure S3: Leave-one-out sensitivity analyses for antioxidant status outcomes (SOD, MDA, and GSH-Px); Figure S4: Forest plots of one-comparison-per-study sensitivity analyses for evaluating the robustness of the meta-analysis results (SOD, MDA, and GSH-Px); Figure S5: Forest plots of one-comparison-per-study sensitivity analyses for evaluating the robustness of the meta-analysis results (ADG, ADFI, and F/G); Figure S6: Funnel plots illustrating trim-and-fill analyses for potential publication bias across all meta-analyzed outcomes; Supplementary Note S1: Reports Not Retrieved.

## References

[1] Hao Y., Xing M., Gu X. (2021). Research progress on oxidative stress and its nutritional regulation strategies in pigs. Animals.

[2] Gessner D.K., Ringseis R., Eder K. (2017). Potential of plant polyphenols to combat oxidative stress and inflammatory processes in farm animals. J. Anim. Physiol. Anim. Nutr..

[3] Liu H., Zhu C., Zhu M., Yuan L., Li S., Gu F., Hu P., Chen S., Cai D. (2024). Alternatives to antibiotics in pig production: Looking through the lens of immunophysiology. Stress Biol..

[4] Panche A.N., Diwan A.D., Chandra S.R. (2016). Flavonoids: An overview. J. Nutr. Sci..

[5] Rice-Evans C.A., Miller N.J., Paganga G. (1996). Structure-antioxidant activity relationships of flavonoids and phenolic acids. Free Radic. Biol. Med..

[6] Dias M., Pinto D., Silva A. (2021). Plant flavonoids: Chemical characteristics and biological activity. Molecules.

[7] Ullah A., Munir S., Badshah S., Khan N., Ghani L., Poulson B., Emwas A.H., Jaremko M. (2020). Important flavonoids and their role as a therapeutic agent. Molecules.

[8] Hassanpour S.H., Doroudi A. (2023). Review of the antioxidant potential of flavonoids as a subgroup of polyphenols and partial substitute for synthetic antioxidants. Avicenna J. Phytomed..

[9] Chen C., Feng F., Qi M., Chen Q., Tang W., Diao H., Hu Z., Qiu Y., Li Z., Chu Y. (2024). Dietary citrus flavonoids improved growth performance and intestinal microbiota of weaned piglets via immune function mediated by TLR2/NF-*κ*B signaling pathway. J. Agric. Food Chem..

[10] Kuhn G., Hennig U., Kalbe C., Rehfeldt C., Ren M.Q., Moors S., Degen G.H. (2004). Growth performance, carcass characteristics and bioavailability of isoflavones in pigs fed soy bean based diets. Arch. Anim. Nutr..

[11] Camargo N.O.T., Cony B.S.L., Silva M.K., Franceschi C.H., Andretta I. (2023). A systematic review and meta-analysis of the effect of phytogenic feed additives on pig performance. Livest. Sci..

[12] Moco S., Martin F.P.J., Rezzi S. (2012). Metabolomics view on gut microbiome modulation by polyphenol-rich foods. J. Proteome Res..

[13] Vanrolleghem W., Tanghe S., Verstringe S., Bruggeman G., Papadopoulos D., Trevisi P., Zentek J., Sarrazin S., Dewulf J. (2019). Potential dietary feed additives with antibacterial effects and their impact on performance of weaned piglets: A meta-analysis. Vet. J..

[14] Page M.J., McKenzie J.E., Bossuyt P.M., Boutron I., Hoffmann T.C., Mulrow C.D., Shamseer L., Tetzlaff J.M., Akl E.A., Brennan S.E. (2021). The PRISMA 2020 statement: An updated guideline for reporting systematic reviews. BMJ.

[15] Kong Y., Tian J., Niu X., Li M., Kong Y., Li R., Chen X., Wang G. (2022). Effects of dietary quercetin on growth, antioxidant capacity, immune response and immune-related gene expression in snakehead fish, Channa argus. Aquac. Rep..

[16] Wang Y., Chen Y., Zhang X., Lu Y., Chen H. (2020). New insights into intestinal oxidative stress damage and the health intervention effects of nutrients: A review. J. Funct. Foods.

[17] Page M.J., Moher D., Bossuyt P.M., Boutron I., Hoffmann T.C., Mulrow C.D., Shamseer L., Tetzlaff J.M., Akl E.A., Brennan S.E. (2021). PRISMA 2020 explanation and elaboration: Updated guidance and exemplars for reporting systematic reviews. BMJ.

[18] Higgins J.P.T., Thomas J., Chandler J., Cumpston M., Li T., Page M.J., Welch V.A. (2019). Cochrane Handbook for Systematic Reviews of Interventions.

[19] Viechtbauer W. (2010). Conducting meta-analyses in R with the metafor package. J. Stat. Softw..

[20] Konstantopoulos S. (2011). Fixed effects and variance components estimation in three-level meta-analysis. Res. Synth. Methods.

[21] Orsini N., Li R., Wolk A., Khudyakov P., Spiegelman D. (2012). Meta-analysis for linear and nonlinear dose-response relations: Examples, an evaluation of approximations, and software. Am. J. Epidemiol..

[22] Crippa A., Orsini N. (2016). Dose-response meta-analysis of differences in means. BMC Med. Res. Methodol..

[23] Egger M., Smith G.D., Schneider M., Minder C. (1997). Bias in meta-analysis detected by a simple, graphical test. BMJ.

[24] Rosenthal R. (1979). The file drawer problem and tolerance for null results. Psychol. Bull..

[25] Hooijmans C.R., Rovers M.M., De Vries R.B.M., Leenaars M., Ritskes-Hoitinga M., Langendam M.W. (2014). SYRCLE’s risk of bias tool for animal studies. BMC Med. Res. Methodol..

[26] Guyatt G.H., Oxman A.D., Vist G.E., Kunz R., Falck-Ytter Y., Alonso-Coello P., Schünemann H.J. (2008). GRADE: An emerging consensus on rating quality of evidence and strength of recommendations. BMJ.

[27] Hooijmans C.R., De Vries R.B., Ritskes-Hoitinga M., Rovers M.M., Leeflang M.M., IntHout J., Wever K.E., Hooft L., De Beer H., Kuijpers T. (2018). Facilitating healthcare decisions by assessing the certainty in the evidence from preclinical animal studies. PLoS ONE.

[28] Liu Y., Xiao Y., Xie J., Peng Y., Li F., Chen C., Li Y., Zhang X., He J., Xiao D. (2022). Dietary supplementation with flavonoids from mulberry leaves improves growth performance and meat quality, and alters lipid metabolism of skeletal muscle in a Chinese hybrid pig. Anim. Feed Sci. Technol..

[29] Mei H.D., Li Y.F., Tian Q., Qu S.F., Ma X.Y., Li Z.M., Gao F.X., Yu M. (2022). Effects of quercetin on growth performance, apparent nutrient digestibility, serum biochemical indices, fecal microbiota and metabolites of weaned piglets. Chin. J. Anim. Nutr..

[30] Park J.H., Sureshkumar S., Kim I.H. (2020). Influences of dietary flavonoid (quercetin) supplementation on growth performance and immune response of growing pigs challenged with Escherichia coli lipopolysaccharide. J. Anim. Sci. Technol..

[31] Degroote J., Vergauwen H., Van Noten N., Wang W., De Smet S., Van Ginneken C., Michiels J. (2019). The effect of dietary quercetin on the glutathione redox system and small intestinal functionality of weaned piglets. Antioxidants.

[32] Che Y., Li L., Kong M., Geng Y., Wang D., Li B., Deng L., Chen G., Wang J. (2024). Dietary supplementation of Astragalus flavonoids regulates intestinal immunology and the gut microbiota to improve growth performance and intestinal health in weaned piglets. Front. Immunol..

[33] Yang M., Yang M. (2024). Effects of quercetin on growth performance, serum immune indices and intestinal digestive enzyme activities of growing pigs. China Feed.

[34] Zhang W., Zhu Q., Zhang Y. (2023). Effects of quercetin on growth performance, intestinal antioxidant and immune-related gene expression in piglets. J. Henan Agric. Univ..

[35] Mao Y., Yang Q., Liu J., Fu Y., Zhou S., Liu J., Ying L., Li Y. (2024). Quercetin increases growth performance and decreases incidence of diarrhea and mechanism of action in weaned piglets. Oxidative Med. Cell. Longev..

[36] Wang L., Hu R., Ma S., Yang X., Gong J., Xiang H., Shi M., Yuan X., Chen L., Zhang H. (2025). Dihydroquercetin attenuated Prevotella copri-caused intestinal injury by modulating gut microbiota and bile acids in weaned piglets. Anim. Nutr..

[37] Ivanova S., Nikolova T., Pirgozliev V., Nenova R. (2024). Effect of dihydroquercetin on performance, back fat thickness and blood biochemical indices in fattening pigs. Sci. Papers Ser. D Anim. Sci..

[38] Ma L., Zhou B., Liu H., Chen S., Zhang J., Wang T., Wang C. (2024). Dietary rutin improves the antidiarrheal capacity of weaned piglets by improving intestinal barrier function, antioxidant capacity and cecal microbiota composition. J. Sci. Food Agric..

[39] Song M., Wang C., Deng D., Liu Z., Chen Q., Gui Y., Lu H., Li Z., Yu M., Ti J. (2022). Effects of mulberry leaf extract on growth performance, apparent nutrient digestibility, antioxidant capacity and immune function of weaned piglets. Chin. J. Anim. Nutr..

[40] Li Y.P., Jiang X.R., Wei Z.X., Cai L., Yin J.D., Li X.L. (2020). Effects of soybean isoflavones on the growth performance, intestinal morphology and antioxidative properties in pigs. Animal.

[41] Li F., Zhu T., Zhang Y., Zhu Y. (2015). Effects of soybean isoflavones on growth performance, immune performance, nutrient digestibility and fecal microflora of weaned piglets. Swine Prod..

[42] Chen H., Wang J., Wang M. (2022). Effects of soybean isoflavones on growth performance, serum biochemical, immune and antioxidant capacities of growing pigs. Chin. J. Anim. Sci..

[43] Lin X.J., Chen F., Jiang S.Q., Jiang Z.Y. (2020). Effects of soybean isoflavones on growth performance, antioxidant function and intestinal mucosal morphology of early weaned piglets. Sci. Agric. Sin..

[44] Payne R.L., Bidner T.D., Southern L.L., Geaghan J.P. (2001). Effects of dietary soy isoflavones on growth, carcass traits, and meat quality in growing-finishing pigs. J. Anim. Sci..

[45] Zhu C., Wu Y., Jiang Z., Zheng C., Wang L., Yang X., Ma X., Gao K., Hu Y. (2015). Dietary soy isoflavone attenuated growth performance and intestinal barrier functions in weaned piglets challenged with lipopolysaccharide. Int. Immunopharmacol..

[46] Li Y., Jiang X., Cai L., Zhang Y., Ding H., Yin J., Li X. (2022). Effects of daidzein on antioxidant capacity in weaned pigs and IPEC-J2 cells. Anim. Nutr..

[47] Cui Y., Tian Z., Wang G., Ma X., Chen W. (2020). Citrus extract improves the absorption and utilization of nitrogen and gut health of piglets. Animals.

[48] Gan H., Lin Q., Xiao Y., Tian Q., Deng C., Xie R., Li H., Ouyang J., Huang X., Shan Y. (2024). Effects of fructus aurantii extract on growth performance, nutrient apparent digestibility, serum parameters, and fecal microbiota in finishing pigs. Animals.

[49] Zhang Q., Cho S., Kim I.H. (2023). The effects of micelle silymarin on growth performance, nutrient utilisation, and blood profiles of weaning piglets. J. Anim. Feed Sci..

[50] Zheng Y., Li Y., Yu B., Luo Y., Huang Z., Zheng P., Mao X., Dai Z., Yu J., Yan H. (2025). Dietary supplementation of grape seed proanthocyanidins improves growth performance, carcass traits, and meat quality in growing-finishing pigs. Anim. Nutr..

[51] Gao Y., Liu J., Yang R., Fan X., Liang C., Shi X., Jin J. (2025). Dietary Baicalin Supplementation Can Enhance the Growth Performance of Weaned Piglets and Maintain the Intestinal Barrier Integrity. Anim. Res. One Health.

[52] Wu H., Peng W., Yu X., Zhang X., Ji F., Shen Q., Lv R. (2025). Effects of Pueraria extracts on growth performance, immune function, and immune-related gene expression of Wuzhishan piglets. Front. Vet. Sci..

[53] Liu J., Qiao Y., Yu B., Luo Y., Huang Z., Mao X., Yu J., Zheng P., Yan H., Li Y. (2023). Functional characterization and toxicological study of proanthocyanidins in weaned pigs. Toxins.

[54] Liang X., Zheng S., Zhou Y., Li J., Zhang Z. (2024). Luteolin, a natural flavonoid, exhibits a protective effect on intestinal injury induced by soybean meal in early-weaned piglets. J. Anim. Sci..

[55] Zou Y., Wei H.K., Xiang Q.H., Wang J., Zhou Y.F., Peng J. (2016). Protective effect of quercetin on pig intestinal integrity after transport stress is associated with regulation oxidative status and inflammation. J. Vet. Med. Sci..

[56] Xiong X., Tan B., Song M., Ji P., Kim K., Yin Y., Liu Y. (2019). Nutritional intervention for the intestinal development and health of weaned pigs. Front. Vet. Sci..

[57] Liao P., Li Y., Li M., Chen X., Yuan D., Tang M., Xu K. (2020). Baicalin alleviates deoxynivalenol-induced intestinal inflammation and oxidative stress damage by inhibiting NF-*κ*B and increasing mTOR signaling pathways in piglets. Food Chem. Toxicol..

[58] Chen J., Huang Z., Cao X., Chen X., Zou T., You J. (2022). Plant-derived polyphenols as Nrf2 activators to counteract oxidative stress and intestinal toxicity induced by deoxynivalenol in swine: An emerging research direction. Antioxidants.

[59] Tang X., Xiong K., Fang R., Li M. (2022). Weaning stress and intestinal health of piglets: A review. Front. Immunol..

[60] Campbell J.M., Crenshaw J.D., Polo J. (2013). The biological stress of early weaned piglets. J. Anim. Sci. Biotechnol..

[61] Yuan D., Wang J., Xiao D., Li J., Liu Y., Tan B., Yin Y. (2020). Eucommia ulmoides flavones as potential alternatives to antibiotic growth promoters in a low-protein diet improve growth performance and intestinal health in weaning piglets. Animals.

[62] Huang C., Wang Y., He X., Jiao N., Zhang X., Qiu K., Piao X., Yin J. (2019). The involvement of NF-*κ*B/P38 pathways in Scutellaria baicalensis extracts attenuating of Escherichia coli K88-induced acute intestinal injury in weaned piglets. Br. J. Nutr..

[63] Johnson R.W., Patience J.F. (2012). Fueling the immune response: What’s the cost?. Feed Efficiency in Swine.

[64] van der Meer Y., Jansman A.J.M., Gerrits W.J.J. (2020). Low sanitary conditions increase energy expenditure for maintenance and decrease incremental protein efficiency in growing pigs. Animal.

[65] Ortega A.D.S.V., Szabó C. (2021). Adverse effects of heat stress on the intestinal integrity and function of pigs and the mitigation capacity of dietary antioxidants: A review. Animals.

[66] Yang D., Wang T., Long M., Li P. (2020). Quercetin: Its main pharmacological activity and potential application in clinical medicine. Oxidative Med. Cell. Longev..

[67] Xiao D., Yuan D., Tan B., Wang J., Liu Y., Tan B. (2019). The role of Nrf2 signaling pathway in Eucommia ulmoides flavones regulating oxidative stress in the intestine of piglets. Oxidative Med. Cell. Longev..

[68] Li E., Li C., Horn N., Ajuwon K.M. (2023). Quercetin attenuates deoxynivalenol-induced intestinal barrier dysfunction by activation of Nrf2 signaling pathway in IPEC-J2 cells and weaned piglets. Curr. Res. Toxicol..

[69] Millecam J., De Clerck L., Govaert E., Devreese M., Gasthuys E., Schelstraete W., Deforce D., De Bock L., Croubels S. (2018). The ontogeny of cytochrome P450 enzyme activity and protein abundance in conventional pigs. Front. Pharmacol..

[70] Buyssens L., Valenzuela A., Prims S., Ayuso M., Thymann T., Van Ginneken C., Van Cruchten S. (2023). Ontogeny of CYP3A and UGT activity in preterm piglets: A translational model for drug metabolism in preterm newborns. Front. Pharmacol..

[71] Kumar S., Pandey A.K. (2013). Chemistry and biological activities of flavonoids: An overview. Sci. World J..

[72] Depeint F., Gee J.M., Williamson G., Johnson I.T. (2002). Evidence for consistent patterns between flavonoid structures and cellular activities. Proc. Nutr. Soc..

[73] Hassan Z.M., Manyelo T.G., Selaledi L., Mabelebele M. (2020). The effects of tannins in monogastric animals with special reference to alternative feed ingredients. Molecules.

[74] Carmona A. (1994). Tannins: Thermostable pigments which complex dietary proteins and inhibit digestive enzymes. Arch. Latinoam. De Nutr..

