# Supplementary material for: Growth Performance and Antioxidant Responses to Dietary Flavonoids in Swine: A Systematic Review and Meta-Analysis with Exploratory Dose–Time Relationships
Source: Antioxidants (Basel). 2026 Aug 7;15(8):980. doi: 10.3390/antiox15080980 (PMC13509563; doi:10.3390/antiox15080980)
Supplement: Supplementary file 1 [file antioxidants-15-00980-s001.zip › Supplementary Materials.pdf]

Supplementary Materials

Growth Performance and Antioxidant Responses to Dietary Flavonoids in Swine: A Systematic Review and Meta-Analysis with Exploratory Dose–Time Relationships

Supplementary Tables

Table S1. PRISMA 2020 Checklist and Location in Manuscript

Section	PRISMA 2020 Item (Abbreviated)	Location in Manuscript
Title	1. Title	Title
Abstract	2. Abstract	Abstract
Introduction	3. Rationale	Introduction
	4. Objectives	Introduction, final paragraph
Methods	5. Eligibility criteria	Materials and Methods, Eligibility Criteria
	6. Information sources	Materials and Methods, Literature Search Strategy
	7. Search strategy	Materials and Methods, Literature Search Strategy; Table S2
	8. Selection process	Materials and Methods, Data Extraction; Figure 1
	9. Data collection	Materials and Methods, Data Extraction
	10. Data items	Materials and Methods, Data Extraction
	11. Study risk of bias	Materials and Methods, Risk of Bias and Certainty of Evidence Assessment
	12. Effect measures	Materials and Methods, Effect Size Calculation and Model Selection
	13. Synthesis methods	Materials and Methods, Statistical Analysis
	14. Reporting bias assessment	Materials and Methods, Publication Bias and Sensitivity Analysis
	15. Certainty assessment	Materials and Methods, Risk of Bias and Certainty of Evidence Assessment; Table S3
Results	16. Study selection	Results, Literature Search and Screening Results; Figure 1
	17. Study characteristics	Results, Literature Search and Screening Results; Table 1
	18. Risk of bias results	Figure 2; Tables S4 and S9
	19. Individual results	Results, Growth Performance and Antioxidant Capacity subsections; Figures 3–5 and 7–9
	20. Synthesis of results	Results, Growth Performance, Dose–Time Meta-Regression, and Antioxidant Capacity subsections; Table 2; Figures 3–9
	21. Reporting biases	Results, Sensitivity Analyses and Publication Bias Assessment; Supplementary Table S5; Supplementary Figures S1 and S6

Continued on next page

Table S1 continued from previous page

Section	Item	Location
	22. Certainty of evidence	Abstract; Materials and Methods, Risk of Bias and Certainty of Evidence Assessment; Supplementary Table S3; Discussion, Limitations of the Present Study
Discussion	23. Discussion	Discussion
Other	24. Registration / Protocol	Materials and Methods, Protocol and Reporting Standard
	25. Support / Funding	End matter, Funding
	26. Competing interests	End matter, Conflicts of Interest
	27. Availability of data, code, and other materials	End matter, Data Availability Statement; analysis code not publicly deposited

Table S2. Detailed Search Strategy Used for Each Database

Database	Search strategy (exact search syntax)
PubMed	((”flavonoid*” OR quercetin OR isoflavone* OR baicalin OR daidzein OR ”soybean isoflavone” OR proanthocyanidin*) AND (pig OR pigs OR swine OR piglet*) AND (”growth performance” OR ”average daily gain” OR ADG OR ”average daily feed intake” OR ADFI OR ”feed conversion ratio” OR FCR OR ”feed-to-gain” OR ”oxidative stress” OR antioxidant OR ”superoxide dismutase” OR SOD OR malondialdehyde OR MDA OR ”glutathione peroxidase” OR GSH-Px)) [Title/Abstract]
Web of Science	TS=(flavonoid* OR quercetin OR isoflavone* OR baicalin OR daidzein OR ”soybean isoflavone” OR proanthocyanidin*) AND TS=(pig OR pigs OR swine OR piglet*) AND TS=(”growth performance” OR ”average daily gain” OR ADG OR ”average daily feed intake” OR ADFI OR ”feed conversion ratio” OR FCR OR ”oxidative stress” OR antioxidant OR SOD OR MDA OR GSH-Px)
Scopus	TITLE-ABS-KEY (flavonoid* OR quercetin OR isoflavone* OR baicalin OR daidzein OR ”soybean isoflavone” OR proanthocyanidin*) AND TITLE-ABS-KEY (pig OR pigs OR swine OR piglet*) AND TITLE-ABS-KEY (”growth performance” OR ADG OR ADFI OR FCR OR antioxidant OR oxidative stress OR SOD OR MDA OR GSH-Px)
CNKI	(黄酮 [flavonoids] OR 槲皮素 [quercetin] OR 异黄酮 [isoflavones] OR 黄芩苷 [baicalin] OR 木犀草素 [luteolin] OR 原花青素 [proanthocyanidins])<br>AND<br>(猪 [pigs] OR 仔猪 [piglets] OR 育肥猪 [finishing pigs] OR 生长猪 [growing pigs])<br>AND<br>(生长性能 [growth performance] OR 平均日增重 [average daily gain] OR 日采食量 [average daily feed intake] OR 料重比 [feed-to-gain ratio] OR 抗氧化 [antioxidant] OR 氧化应激 [oxidative stress] OR 超氧化物歧化酶 [superoxide dismutase] OR 谷胱甘肽过氧化物酶 [glutathione peroxidase] OR 丙二醛 [malondialdehyde])

Note: The original Chinese search terms were retained to ensure reproducibility; their English translations are provided in square brackets.

Table S3. GRADE Certainty Assessment and Reasons for Downgrading

Outcome	k	Risk of Bias	Inconsistency	Indirectness	Imprecision	Publication Bias	Certainty	Downgrading rationale
ADG	67	Not serious*	Serious	Not serious	Not serious	Serious	Low	Downgraded one level for inconsistency ($I^2 = 51.85\%$) and one level for potential publication bias because Egger's test indicated funnel-plot asymmetry ($P = 0.030$).
ADFI	67	Not serious*	Serious	Not serious	Not serious	Not serious	Moderate	Downgraded one level for inconsistency ($I^2 = 59.30\%$). Egger's test did not indicate funnel-plot asymmetry ($P = 0.557$).
F/G	67	Not serious*	Not serious	Not serious	Not serious	Serious	Moderate	Downgraded one level for potential publication bias because Egger's test indicated funnel-plot asymmetry ($P = 0.0068$).
SOD	41	Not serious*	Serious	Not serious	Not serious	Not serious	Moderate	Downgraded one level for substantial inconsistency ($I^2 = 97.29\%$). Egger's test did not indicate funnel-plot asymmetry ($P = 0.352$).
MDA	38	Not serious*	Serious	Not serious	Not serious	Not serious	Moderate	Downgraded one level for substantial inconsistency ($I^2 = 97.83\%$). Egger's test did not indicate funnel-plot asymmetry ($P = 0.788$).
GSH-Px	35	Not serious*	Serious	Not serious	Not serious	Not serious	Moderate	Downgraded one level for substantial inconsistency ($I^2 = 96.47\%$). Egger's test did not indicate funnel-plot asymmetry ($P = 0.836$).

Certainty assessment was conducted using an adapted GRADE approach for animal nutrition studies. Evidence was initially rated as high certainty because all included studies were randomized controlled trials. Downgrading decisions were based on inconsistency ($I^2 > 50\%$) and potential publication bias indicated by Egger's test ($P < 0.05$). The value of k represents the number of effect sizes included in the primary three-level meta-analysis. Publication-bias diagnostics were conducted using datasets retaining one comparison per study. *Although several SYRCLE domains were rated as unclear risk, these judgments mainly reflected insufficient reporting rather than confirmed methodological flaws. Therefore, risk of bias was not downgraded in the adapted GRADE assessment.

Table S4. Summary of Risk of Bias Assessment Using the SYRCLE Tool

SYRCLE domain	Low risk, n (%)	Unclear risk, n (%)	High risk, n (%)
Random sequence generation	28 (100.0)	0 (0.0)	0 (0.0)
Baseline characteristics	28 (100.0)	0 (0.0)	0 (0.0)
Allocation concealment	0 (0.0)	28 (100.0)	0 (0.0)
Random housing	10 (35.7)	18 (64.3)	0 (0.0)
Blinding of caregivers/researchers	0 (0.0)	28 (100.0)	0 (0.0)
Random outcome assessment	13 (46.4)	15 (53.6)	0 (0.0)
Blinding of outcome assessor	0 (0.0)	28 (100.0)	0 (0.0)
Incomplete outcome data	28 (100.0)	0 (0.0)	0 (0.0)
Selective reporting	28 (100.0)	0 (0.0)	0 (0.0)
Other bias	27 (96.4)	0 (0.0)	1 (3.6)

Values are numbers of studies with percentages in parentheses. Percentages were calculated using 28 included studies as the denominator. The domain-level counts correspond to the detailed study-level judgments reported in Table S9 and are visualized in Figure 2 of the main manuscript.

Table S5. Publication-Bias Diagnostics and Trim-and-Fill Analyses Based on One Comparison per Study

Outcome	Studies	Egger P	k_0	Original SMD (95% CI)	Adjusted SMD (95% CI)
Panel A. growth performance outcomes					
ADG	26	0.030	5	0.665 [0.378, 0.951]<br>$P < 0.001$	0.441 [0.107, 0.774]<br>$P = 0.010$
ADFI	26	0.557	0	0.074 [−0.213, 0.361]<br>$P = 0.611$	0.074 [−0.213, 0.361]<br>$P = 0.611$
F/G	26	0.0068	3	−0.707 [−0.946, −0.468]<br>$P < 0.001$	−0.599 [−0.876, −0.321]<br>$P < 0.001$
Panel B. Antioxidant outcomes					
SOD	15	0.352	0	1.301 [0.673, 1.930]<br>$P < 0.001$	1.301 [0.673, 1.930]<br>$P < 0.001$
MDA	14	0.788	0	−1.159 [−1.815, −0.503]<br>$P < 0.001$	−1.159 [−1.815, −0.503]<br>$P < 0.001$
GSH-Px	12	0.836	0	0.721 [0.414, 1.029]<br>$P < 0.001$	0.721 [0.414, 1.029]<br>$P < 0.001$

SMD, standardized mean difference; CI, confidence interval; k_0, number of potentially missing studies estimated using the trim-and-fill procedure. Publication-bias analyses were based on datasets retaining one comparison per study. Egger's tests indicated possible funnel-plot asymmetry for ADG and F/G, but not for ADFI, SOD, MDA, or GSH-Px. The trim-and-fill procedure imputed five potentially missing studies for ADG and three for F/G. On the original mean-difference scale, Egger's test was not significant for ADG ($P = 0.447$) or ADFI ($P = 0.963$) but remained significant for F/G ($P = 0.010$). Although the adjusted effect estimates for ADG and F/G were smaller in magnitude, both remained statistically significant. No additional studies were imputed for ADFI, SOD, MDA, or GSH-Px. Trim-and-fill findings should be interpreted cautiously, particularly for antioxidant outcomes with substantial heterogeneity.

Table S6. Stage-Specific Three-Level Meta-Regression of Total Additive Dose and Intervention Duration for growth performance outcomes

**Stage**	**Outcome**	**Effect sizes**	**Studies**	**Dose coefficient per 100 mg/kg**	**Duration coefficient per day**	**Joint P**	**Pseudo-R^2**
Weaned	ADG	35	14	0.0922 [0.0332, 0.1513], $P = 0.002$	0.0016 [−0.0108, 0.0141], $P = 0.797$	0.009	49.88%
Weaned	F/G	35	14	−0.0299 [−0.0963, 0.0364], $P = 0.376$	−0.0041 [−0.0214, 0.0131], $P = 0.637$	0.615	0.00%
Growing	ADG	7	3	Not estimable	Not estimable	–	–
Growing	F/G	7	3	Not estimable	Not estimable	–	–
Finishing	ADG	12	4	0.1662 [0.0630, 0.2694], $P = 0.002$	0.0170 [0.0016, 0.0324], $P = 0.031$	0.004	100.00%
Finishing	F/G	12	4	−0.1634 [−0.2670, −0.0598], $P = 0.002$	−0.0229 [−0.0384, −0.0074], $P = 0.004$	0.002	100.00%

Dose and intervention duration were entered simultaneously into three-level random-effects meta-regression models, with effect sizes nested within studies. Dose coefficients represent the change in Hedges' g per 100 mg/kg increase in the reported total additive dose, whereas duration coefficients represent the change per additional intervention day. Joint P values refer to the omnibus test of dose and duration moderators. Pseudo-R^2 represents the proportional reduction in the summed random-effect variance relative to the corresponding intercept-only model. Models for growing pigs could not be reliably estimated because only seven effect sizes from three independent studies were available. Because the reported dose represented the total amount of the administered product rather than analytically verified active flavonoid content, these associations should be considered exploratory.

Table S7. Complete Subgroup Analysis Results for Growth-Performance and Antioxidant Outcomes

Outcome	Moderator	Subgroup	k	Studies	SMD (95% CI)	P	P_{between}	I^2 (%)
Panel A. growth performance outcomes								
ADG	Class	Flavonols	23	10	0.491 [0.132, 0.850]	0.007	0.388	47.99
ADG	Class	Isoflavones	23	9	0.605 [−0.096, 1.306]	0.091	0.388	65.69
ADG	Class	Flavanones	3	2	1.045 [0.304, 1.787]	0.006	0.388	0.00
ADG	Class	Proanthocyanidins	8	2	1.641 [0.902, 2.380]	< 0.001	0.388	36.28
ADG	Stage	Finishing	14	5	0.722 [−0.068, 1.511]	0.073	0.889	67.93
ADG	Stage	Weaned	46	18	0.766 [0.380, 1.153]	< 0.001	0.889	56.93
ADG	Stage	Growing	7	3	0.513 [0.132, 0.894]	0.008	0.889	0.00
ADFI	Class	Flavonols	23	10	−0.001 [−0.362, 0.360]	0.995	0.826	42.17
ADFI	Class	Isoflavones	23	9	0.161 [−0.585, 0.908]	0.672	0.826	69.13
ADFI	Class	Flavanones	3	2	1.001 [−0.583, 2.585]	0.216	0.826	67.76
ADFI	Class	Proanthocyanidins	8	2	0.526 [−1.285, 2.337]	0.569	0.826	81.66
ADFI	Stage	Finishing	14	5	−0.150 [−1.092, 0.793]	0.756	0.447	73.24
ADFI	Stage	Weaned	46	18	0.350 [−0.030, 0.731]	0.071	0.447	57.27
ADFI	Stage	Growing	7	3	0.028 [−0.348, 0.404]	0.884	0.447	0.00
F/G	Class	Flavonols	23	10	−0.594 [−0.847, −0.341]	< 0.001	0.014	11.54
F/G	Class	Isoflavones	23	9	−0.565 [−0.976, −0.154]	0.007	0.014	29.95
F/G	Class	Flavanones	3	2	−0.844 [−1.568, −0.120]	0.022	0.014	0.00
F/G	Class	Proanthocyanidins	8	2	−1.768 [−2.406, −1.130]	< 0.001	0.014	42.22
F/G	Stage	Finishing	14	5	−0.993 [−1.501, −0.485]	< 0.001	0.406	45.28
F/G	Stage	Weaned	46	18	−0.707 [−0.992, −0.422]	< 0.001	0.406	35.90
F/G	Stage	Growing	7	3	−0.331 [−0.709, 0.048]	0.087	0.406	0.00
Panel B. Antioxidant outcomes								
SOD	Class	Flavonols	6	3	1.068 [0.064, 2.072]	0.037	0.281	96.49
SOD	Class	Isoflavones	17	6	0.683 [0.021, 1.346]	0.043	0.281	97.66
SOD	Stage	Finishing	10	3	1.244 [−0.089, 2.576]	0.067	0.146	98.16
SOD	Stage	Weaned	28	11	0.921 [0.530, 1.311]	< 0.001	0.146	96.31
SOD	Sample type	Serum	24	8	1.073 [0.434, 1.713]	0.001	0.227	97.20
SOD	Sample type	Jejunum	2	2	2.246 [1.881, 2.610]	< 0.001	0.227	33.23
SOD	Sample type	Plasma	10	6	0.214 [−0.377, 0.805]	0.478	0.227	94.47
SOD	Sample type	Liver	3	3	1.286 [0.612, 1.959]	< 0.001	0.227	94.15
SOD	Tissue group	Blood	34	14	0.774 [0.279, 1.268]	0.002	0.313	97.13
SOD	Tissue group	Intestinal tissue	4	4	1.696 [1.089, 2.303]	< 0.001	0.313	91.99
SOD	Tissue group	Liver	3	3	1.286 [0.612, 1.959]	< 0.001	0.313	94.15

Continued on next page

Table S7 continued from previous page

Outcome	Moderator	Subgroup	k	Studies	SMD (95% CI)	P	P_{between}	I^2 (%)
MDA	Class	Flavonols	6	3	−1.658 [−1.802, −1.514]	< 0.001	0.548	0.00
MDA	Class	Isoflavones	17	6	−1.063 [−2.156, 0.030]	0.057	0.548	98.12
MDA	Stage	Finishing	10	3	−1.426 [−2.089, −0.763]	< 0.001	< 0.001	95.68
MDA	Stage	Weaned	25	10	−0.564 [−1.051, −0.077]	0.023	< 0.001	95.72
MDA	Sample type	Serum	21	7	−0.679 [−1.638, 0.280]	0.165	0.313	97.98
MDA	Sample type	Jejunum	2	2	−0.321 [−1.281, 0.639]	0.512	0.313	93.04
MDA	Sample type	Plasma	10	6	−1.122 [−2.229, −0.015]	0.047	0.313	97.44
MDA	Sample type	Liver	3	3	−0.350 [−0.515, −0.186]	< 0.001	0.313	26.50
MDA	Tissue group	Blood	31	13	−0.884 [−1.609, −0.158]	0.017	0.065	97.89
MDA	Tissue group	Intestinal tissue	4	4	−0.989 [−1.840, −0.138]	0.023	0.065	96.60
MDA	Tissue group	Liver	3	3	−0.350 [−0.515, −0.186]	< 0.001	0.065	26.50
GSH-Px	Class	Flavonols	5	2	0.531 [−0.359, 1.421]	0.242	0.234	94.55
GSH-Px	Class	Isoflavones	17	6	0.635 [0.226, 1.043]	0.002	0.234	95.53
GSH-Px	Stage	Finishing	10	3	0.933 [−0.037, 1.903]	0.059	0.476	97.84
GSH-Px	Stage	Weaned	22	8	0.646 [0.313, 0.979]	< 0.001	0.476	94.71
GSH-Px	Sample type	Serum	19	5	0.643 [0.058, 1.228]	0.031	0.352	96.41
GSH-Px	Sample type	Plasma	10	6	0.509 [0.031, 0.987]	0.037	0.352	93.71
GSH-Px	Sample type	Liver	3	3	1.021 [−0.364, 2.406]	0.148	0.352	98.74
GSH-Px	Tissue group	Blood	29	11	0.585 [0.212, 0.958]	0.002	0.348	95.55
GSH-Px	Tissue group	Intestinal tissue	3	3	1.278 [−0.067, 2.623]	0.063	0.348	97.67
GSH-Px	Tissue group	Liver	3	3	1.021 [−0.364, 2.406]	0.148	0.348	98.74

SMD, standardized mean difference; CI, confidence interval; k, number of effect sizes; Studies, number of independent studies. The P column tests the pooled effect within each subgroup, whereas P_{between} tests the difference among levels of the corresponding moderator. Subgroup estimates based on small numbers of independent studies should be interpreted cautiously. Some subgroup totals are smaller than the overall totals because categories with insufficient data for reliable model estimation were not reported.

Table S8. Leave-One-Study-Out Sensitivity Analysis Based on Three-Level Models

Outcome	Three-level Model SMD	Leave-one-study-out Range	Stability
ADG	0.719	0.650–0.784	Stable
ADFI	0.217	0.127–0.267	Stable
F/G	−0.708	−0.747–−0.651	Stable
SOD	0.907	0.745–1.020	Stable
MDA	−0.989	−1.154–−0.763	Stable
GSH-Px	0.666	0.549–0.750	Stable

Leave-one-study-out sensitivity analyses were performed using three-level random-effects models to evaluate whether any individual study substantially influenced the pooled estimates. The ranges represent the minimum and maximum pooled SMDs obtained after sequentially omitting each study. All leave-one-study-out estimates retained the direction of the corresponding primary effect. ADG, F/G, SOD, MDA, and GSH-Px remained statistically significant after the sequential omission of individual studies, whereas ADFI remained non-significant.

Table S9. Detailed Risk of Bias Judgments for Individual Included Studies Based on the SYRCLE Tool

	Selection Bias			Performance Bias		Detection Bias		Attrition	Reporting	Other
**Study ID**	**Q1**	**Q2**	**Q3**	**Q4**	**Q5**	**Q6**	**Q7**	**Q8**	**Q9**	**Q10**
1. Liu_22	L	L	U	U	U	L	U	L	L	L
2. Mei_22	L	L	U	U	U	L	U	L	L	L
3. Park_20	L	L	U	U	U	L	U	L	L	L
4. Degroote_19	L	L	U	L	U	L	U	L	L	L
5. Che_24	L	L	U	U	U	U	U	L	L	L
6. Yang_24	L	L	U	U	U	U	U	L	L	L
7. Zhang_23	L	L	U	U	U	U	U	L	L	L
8. Mao_24	L	L	U	U	U	L	U	L	L	L
9. Liu_23a	L	L	U	U	U	U	U	L	L	H
10. Wang_25	L	L	U	U	U	U	U	L	L	L
11. Ivanova_24	L	L	U	U	U	L	U	L	L	L
12. Liang_24	L	L	U	L	U	L	U	L	L	L
13. Ma_24	L	L	U	L	U	L	U	L	L	L
14. Song_22	L	L	U	L	U	U	U	L	L	L
15. Zou_16	L	L	U	U	U	L	U	L	L	L
16. Li_20	L	L	U	L	U	L	U	L	L	L
17. Li_15	L	L	U	U	U	U	U	L	L	L
18. Chen_22	L	L	U	U	U	U	U	L	L	L
19. Lin_20	L	L	U	L	U	U	U	L	L	L
20. Payne_01	L	L	U	U	U	U	U	L	L	L
21. Zhu_15	L	L	U	L	U	L	U	L	L	L
22. Li_22	L	L	U	L	U	U	U	L	L	L
23. Cui_20	L	L	U	U	U	L	U	L	L	L
24. Gan_24	L	L	U	U	U	L	U	L	L	L
25. Zhang_24	L	L	U	L	U	U	U	L	L	L
26. Zheng_25	L	L	U	U	U	U	U	L	L	L
27. Gao_25	L	L	U	L	U	U	U	L	L	L
28. Wu_25	L	L	U	U	U	U	U	L	L	L

Legend: L, low risk of bias; U, unclear risk of bias; H, high risk of bias.
Selection bias: Q1, sequence generation; Q2, baseline characteristics; Q3, allocation concealment.
Performance bias: Q4, random housing; Q5, blinding of caregivers and researchers.
Detection bias: Q6, random outcome assessment; Q7, blinding of outcome assessors.
Attrition bias: Q8, incomplete outcome data.
Reporting bias: Q9, selective outcome reporting.
Other bias: Q10, other sources of bias.

Table S10. Sensitivity Analysis Using One Comparison per Study to Evaluate Dependent Effect Sizes

Outcome	Primary Three-level Model	One-comparison-per-study Model	Conclusion
ADG	0.719	0.665	Consistent
ADFI	0.217	0.074	Consistent
F/G	−0.708	−0.707	Consistent
SOD	0.907	1.301	Consistent
MDA	−0.989	−1.159	Consistent
GSH-Px	0.666	0.721	Consistent

The primary analyses were performed using three-level random-effects models that accounted for dependent effect sizes nested within studies. Sensitivity analyses were conducted by retaining one comparison per study. Consistent indicates that the direction and statistical-significance classification of the pooled effects remained unchanged. ADG, F/G, SOD, MDA, and GSH-Px were statistically significant in both analyses, whereas ADFI remained non-significant.

Table S11. Original Reported Doses and Standardized Supplementation Doses Used in the Dose–Time Meta-Regression Analyses

Study	Original reported dose	Standardized dose (mg/kg diet)
Degroote (2019)	100, 300, 900 mg/kg	100, 300, 900
Gao (2025)	0.05%, 0.10%, 0.15%	500, 1000, 1500
Li (2015)	5, 10, 15 mg/kg	5, 10, 15
Li (2022)	25, 50, 100 mg/kg	25, 50, 100
Lin (2020)	10, 20, 40, 80 mg/kg	10, 20, 40, 80
Liu (2022)	200, 400, 800, 1600 mg/kg	200, 400, 800, 1600
Liu (2023)	30, 300, 600, 1200 mg/kg	30, 300, 600, 1200
Ma (2024)	500 mg/kg	500
Mao (2024)	250, 500, 750 mg/kg	250, 500, 750
Mei (2022)	200, 400 mg/kg	200, 400
Park (2020)	0.1%	1000
Song (2022)	1000 g/t	1000
Wu (2025)	400, 600, 800 mg/kg	400, 600, 800
Yang (2024)	50, 150, 450 mg/kg	50, 150, 450
Zhang (2023)	200, 400 mg/kg	200, 400
Zhang (2024)	0.05%, 0.10%	500, 1000
Chen (2022)	10, 20, 40 mg/kg	10, 20, 40
Gan (2024)	500, 1000 mg/kg	500, 1000
Ivanova (2024)	100, 200 mg/kg	100, 200
Zheng (2025)	15, 30, 60, 120 mg/kg	15, 30, 60, 120
Zhu (2015)	40 mg/kg	40

Notes: Only studies for which the reported supplementation dose could be reliably converted to mg/kg diet were included in the dose–time meta-regression analyses. Percentage-based supplementation levels were converted assuming a weight/weight feed basis. The standardized dose represents the total amount of the supplemented product and should therefore be interpreted as an indicator of supplementation intensity rather than analytically verified active flavonoid content. Studies with insufficient information for reliable dose conversion remained eligible for the overall meta-analysis but were excluded from the dose–time meta-regression analyses.

Supplementary Figures

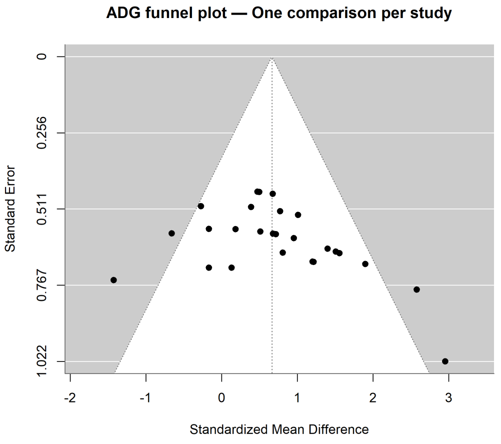

(a) Funnel plot for ADG

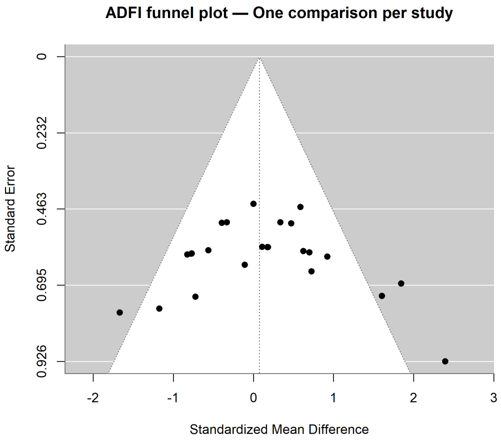

(b) Funnel plot for ADFI

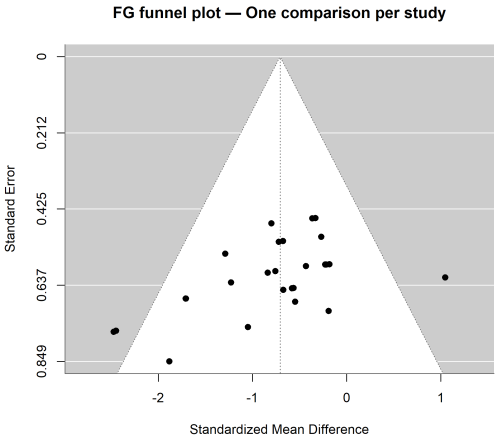

(c) Funnel plot for F/G

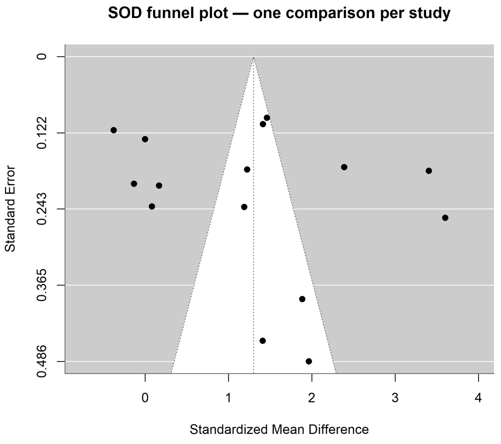

(d) Funnel plot for SOD

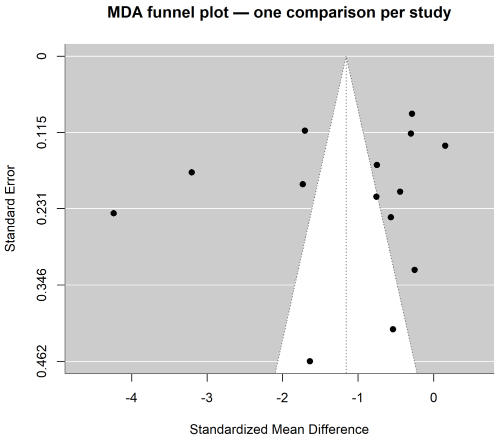

(e) Funnel plot for MDA

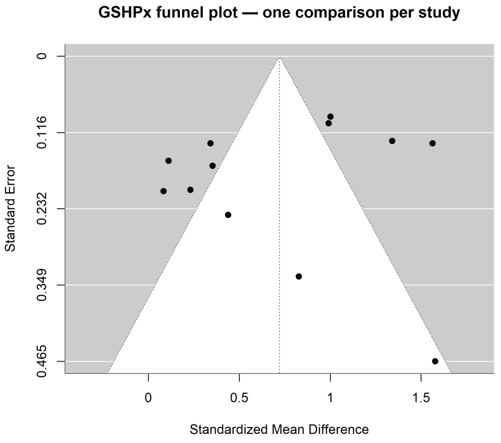

(f) Funnel plot for GSH-Px

Figure S1. Funnel plots for publication-bias assessment based on datasets retaining one comparison per study.

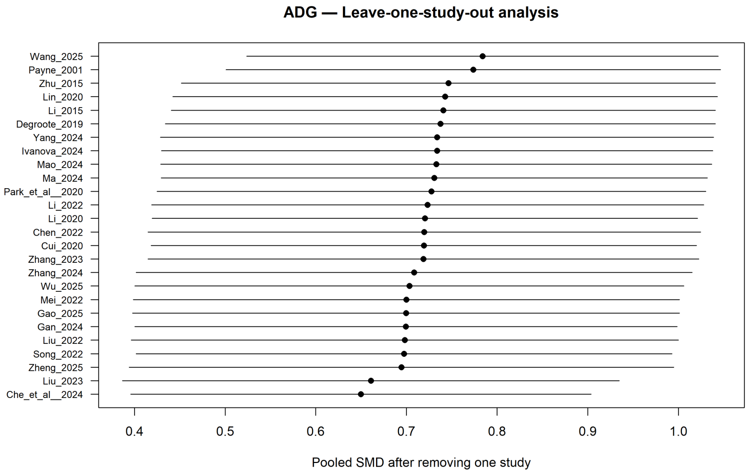

(a) ADG

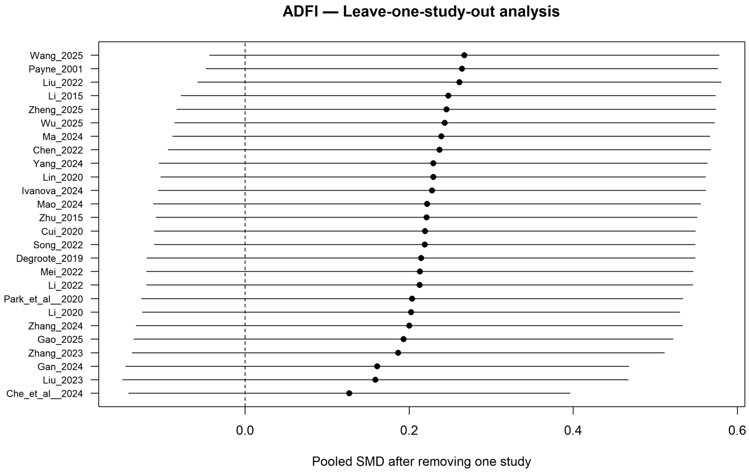

(b) ADFI

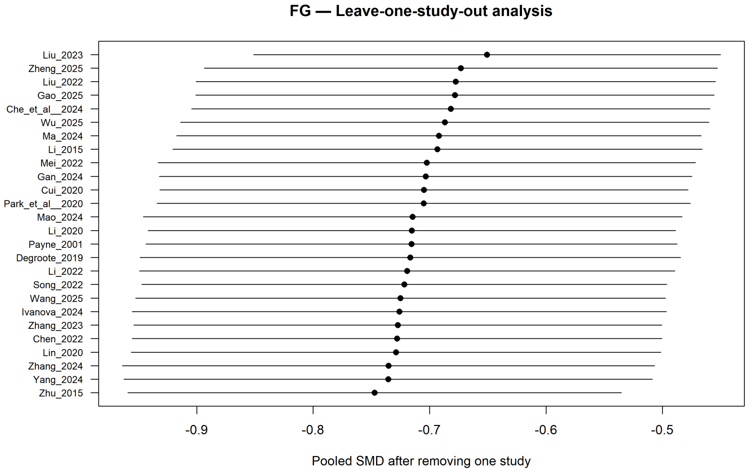

(c) F/G

Figure S2. Leave-one-out sensitivity analyses for growth performance outcomes (ADG, ADFI, and F/G).

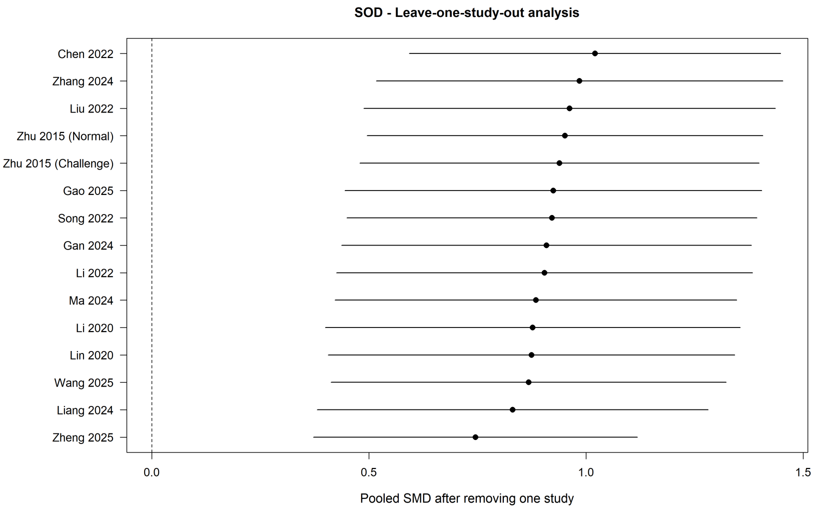

(a) SOD

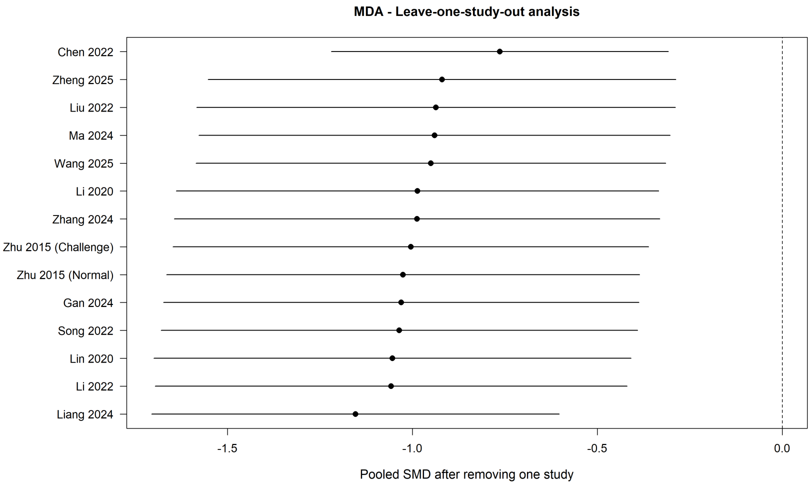

(b) MDA

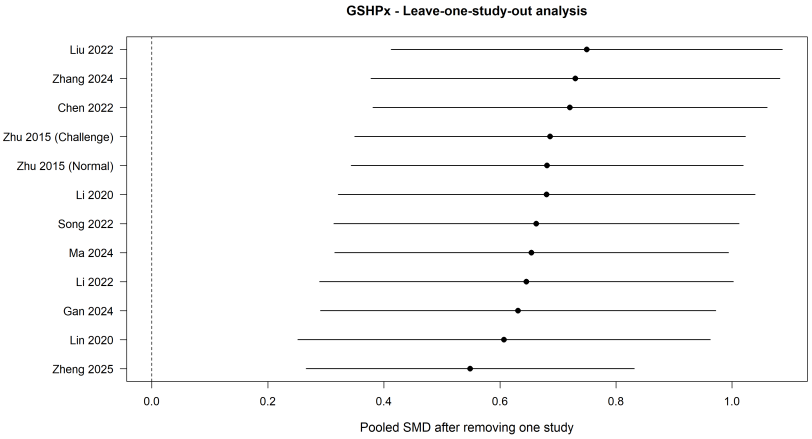

(c) GSH-Px

Figure S3. Leave-one-out sensitivity analyses for antioxidant status outcomes (SOD, MDA, and GSH-Px).

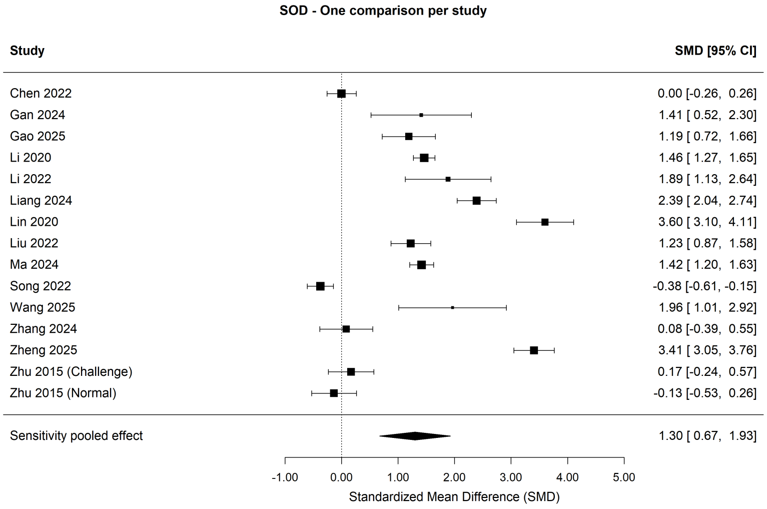

(a) SOD

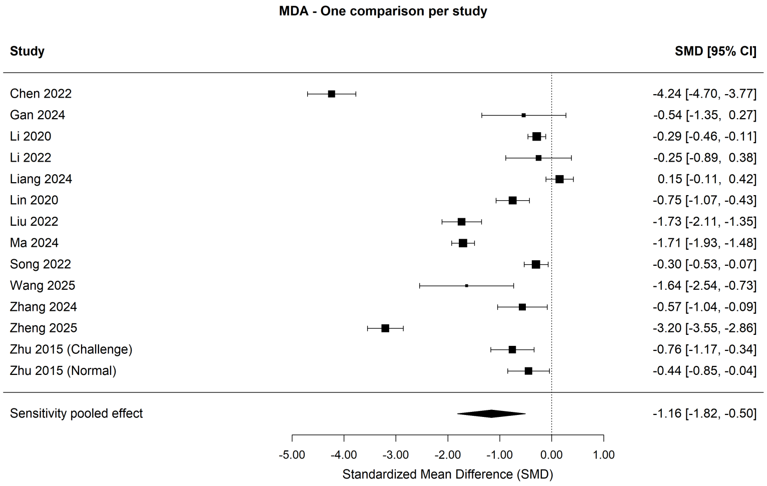

(b) MDA

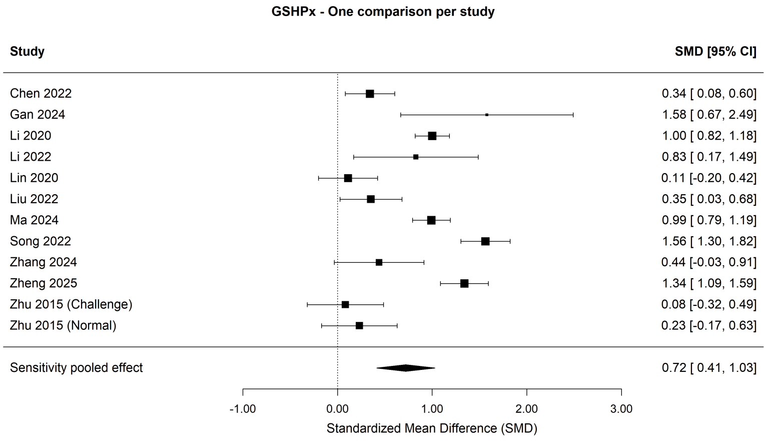

(c) GSH-Px

Figure S4. Forest plots of one-comparison-per-study sensitivity analyses for evaluating the robustness of the meta-analysis results (SOD, MDA, and GSH-Px).

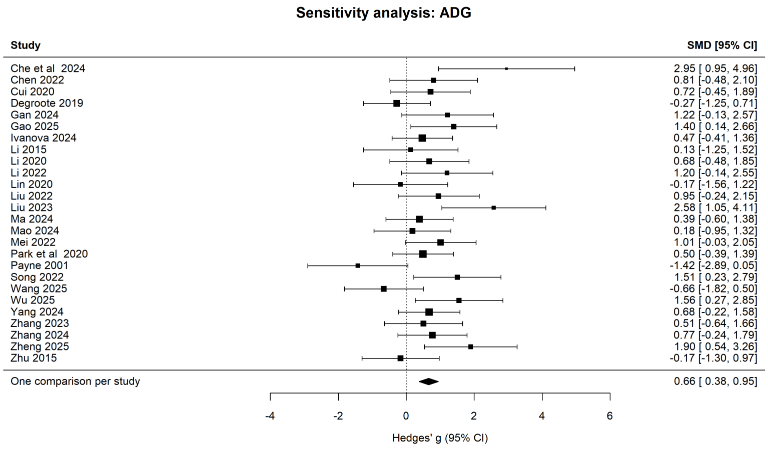

(a) ADG

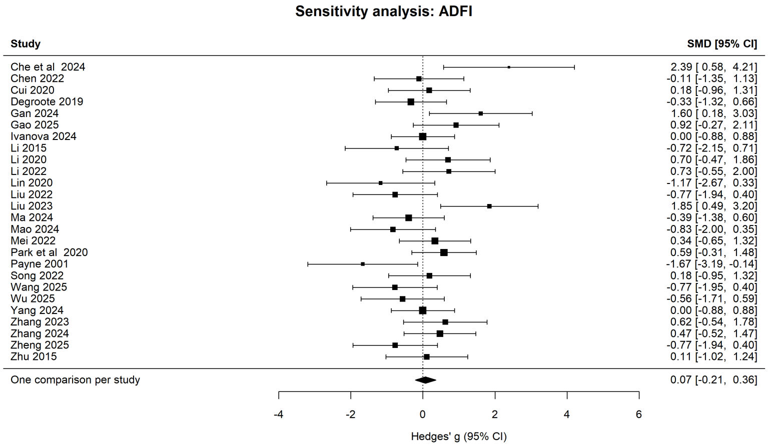

(b) ADFI

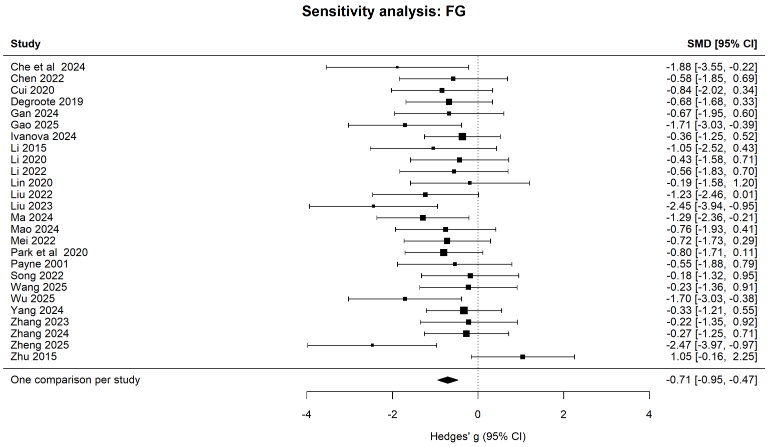

(c) F/G

Figure S5. Forest plots of one-comparison-per-study sensitivity analyses for evaluating the robustness of the meta-analysis results (ADG, ADFI, and F/G).

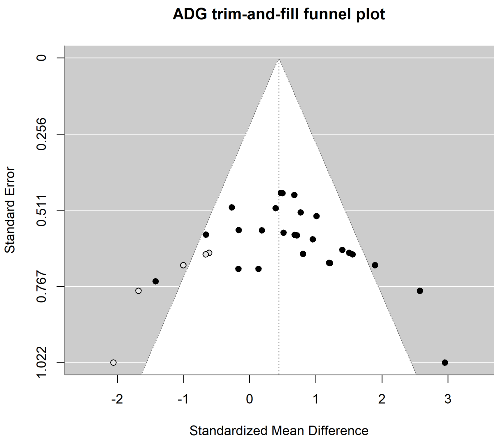

(a) Funnel plot for ADG

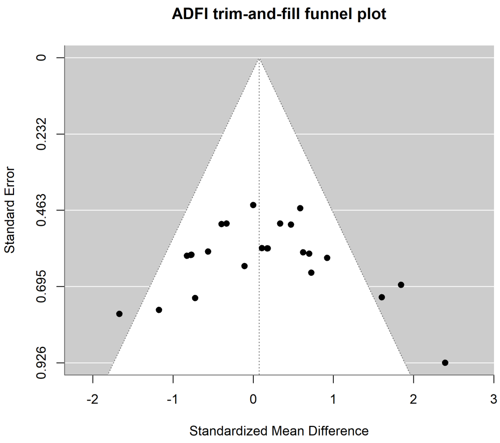

(b) Funnel plot for ADFI

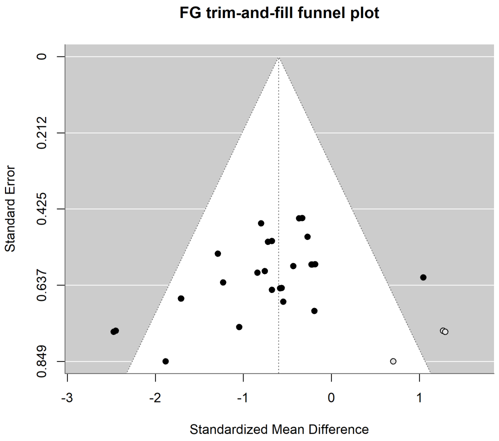

(c) Funnel plot for F/G

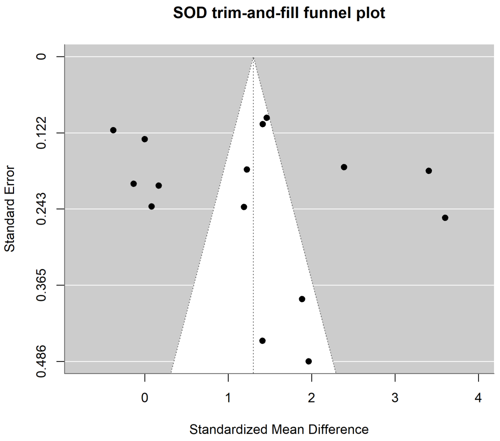

(d) Funnel plot for SOD

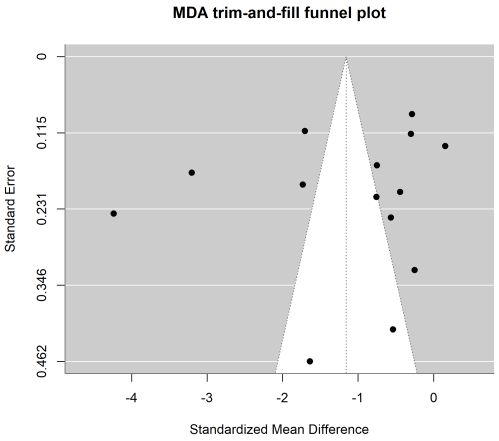

(e) Funnel plot for MDA

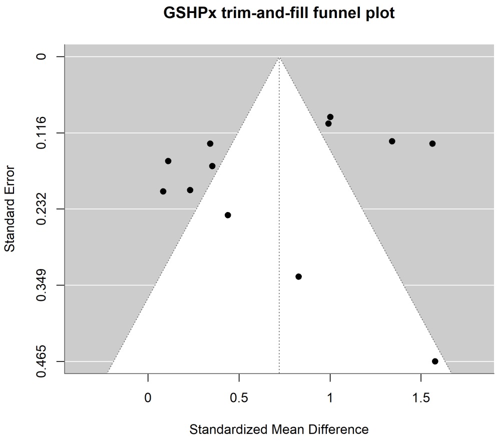

(f) Funnel plot for GSH-Px

Figure S6. Funnel plots illustrating trim-and-fill analyses for potential publication bias across all meta-analyzed outcomes.

Supplementary Note S1. Reports Not Retrieved

Fifteen potentially eligible reports could not be retrieved after additional retrieval attempts and were therefore not assessed for eligibility. Because this review was conducted retrospectively and complete bibliographic identifiers for these reports were not retained in the reconstructed screening

records, an accurate study-level list could not be provided without a risk of misidentification. Their omission has been acknowledged as a potential source of selection bias in the revised manuscript.
